# Gas Sorption Characterization of Porous Materials Employing a Statistical Theory for Bethe Lattices

**DOI:** 10.1021/acs.jpca.4c02185

**Published:** 2024-05-24

**Authors:** E. S. Kikkinides, D. Enke, R. Valiullin

**Affiliations:** † Department of Chemical Engineering, Aristotle University of Thessaloniki, 54124 Thessaloniki, Greece; ‡ Faculty of Chemistry and Mineralogy, Leipzig University, 04103 Leipzig, Germany; § Faculty of Physics and Earth System Sciences, Leipzig University, 04103 Leipzig, Germany

## Abstract

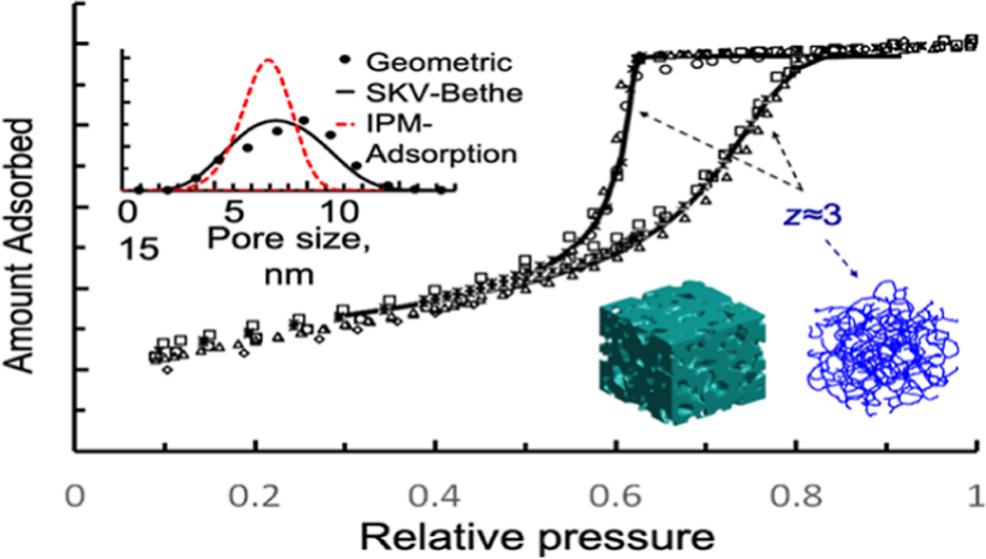

In the present work, a recently developed statistical theory for adsorption and desorption processes in mesoporous solids, modeled by random Bethe lattices, has been applied to obtain pore size distributions and interpore connectivity from sorption isotherms in real random porous materials, employing a robust and validated methodology. Using the experimental adsorption–desorption N_2_ isotherms at 77.4 K on Vycor glass, a porous material with random pore structure, we demonstrate the solution of the inverse problem resulting in extracted pore size distribution and interpore connectivity, notably different from the predictions of earlier theories. The results presented are corroborated by the analysis of 3D digital images of reconstructed Vycor porous glass, showing excellent agreement between the predictions of geometric analysis and the new statistical theory.

## 1 Introduction

Mesoporous materials (pore sizes ranging from 2 to 50 nm) are widely used in various technological applications, including catalysis, gas and liquid separations, energy storage, controlled drug delivery, etc. In all these applications, improvement in process performance can be achieved with a purposeful design of the pore space architecture.^1,2^ The latter relies on the existence of accurate and reliable pore structural characterization methods. Direct imaging of the pore structure, e.g., using electron microscopy [scanning electron microscopy and/or transmission electron microscopy (TEM)], is very challenging for materials with pore sizes in the lower mesopore range caused by limitations in spatial resolution. On the other hand, this method is accompanied by technical difficulties in sample preparation and poor statistics, with the latter being especially important when materials possess substantial structural disorder and anisotropy.^3–7^ Due to these challenges, probing the phase-transition behavior of confined matter, such as done in gas physisorption or thermoporometry, remains the most popular method for structural characterization of mesoporous materials.^8^ Mercury intrusion is also often included into this family for methodological similarities.^9^

Among these methods, gas physisorption is most commonly used and routinely applied for the characterization of mesoporous solids.^10,11^ Progress done over the last decades in the theoretical description of confined fluids allowed to establish very accurate relationships between their physical state at different thermodynamic conditions and the geometric and energetic properties of the porous solids, such as characteristic pore size, pore shape, specific surface area, and surface chemistry. These theories capture the formation of mono- or multilayers of the guest gas molecules at the adsorbent surfaces at low pressures and the occurrence of the capillary-condensation and capillary-evaporation transitions at pressures lower than the bulk saturation pressure. Starting from the original Kelvin–Cohan model,^12,13^ further improvements have been accomplished by Derjaguin^14^ and Broekhoff and De Boer (BDB method),^15,16^ with various modifications,^17–20^ to account for the influence of surface forces on the equilibrium and stability of adsorption films. More recently, the BDB method has been further modified to include the solid–fluid interaction at the atomic level and to employ thermal activation–evaporation mechanisms to account for activated condensation and cavitation.^21–24^ Alternatively, adsorption–desorption isotherms were obtained by Monte Carlo simulations and non-local density functional theory (NLDFT) on simple pore units such as cylinders or slits of various sizes.^25–27^ Predictions of these theories, namely, transition kernels for simple pore geometries, are found to be in very good agreement with the experimental data obtained in ordered mesoporous solids and have been shown to accurately describe the occurrence of the hysteresis between capillary-adsorption and capillary-desorption, the locations of the closure points, and the isotherm shapes.^27^

Despite the continuous improvement in understanding the phase behavior in single pores, little has been done in improving the theoretical models to include the structural representation of the porous materials in a consistent manner. Thus, one of the main properties of interest for mesoporous solids, the pore size distribution function (PSD), is still routinely assessed using the BJH (Barett, Joyner, and Halenda^28^) method or alike. These methods are constructed around the basic assumption that the phase changes occurring in different pores are not correlated, as described in the Everett’s independent domain theory.^29,30^ We will conventionally refer to these models under the general acronym IPM, for independent pore models. The interpore, or simply, pore connectivity, inherent in real materials and leading to cooperative effects in phase transitions, is completely lacking in IPM. Accordingly, a PSD extracted in this way for a real porous solid represents the size distribution for a collection of individual pores best matching one of the experimental sorption transitions, either adsorption or desorption. Because these cooperativity effects affect the experimentally measured adsorption and desorption transitions differently, as a rule, the PSDs obtained from adsorption and desorption branches do not coincide.^13^ Moreover, the ignorance of the pore connectivity renders these PSDs being different from the real PSD of the material under study. The latter, in particular, finds an increasing evidence from computer simulation studies.^31–37^

Capillary-condensation and evaporation transitions have been studied using lattice fluid models, since they can be easily employed in simple as well as in complex pore geometries, or pore networks.^33,34,38–40^ In particular, insight into the mechanisms controlling the transition behavior beyond that of IPM has been gained by considering ink-bottle pore systems, representing the elementary units of statistically disordered materials.^41,42^ Linear ink-bottle chains and networks of pores with different pore sizes have been addressed by numerical simulations.^43–47^ On the other hand, theoretical models of capillary phenomena were mostly concerned with the application of percolation theory during desorption, in which the connectivity effects were modeled employing the loop-less Bethe lattices,^48–54^ while connectivity effects during adsorption have been ignored, with a few exceptions.^45,55,56^

Considering the established mechanisms of how the cooperativity phenomena in phase transitions emerge locally in the ink-bottle systems, Valiullin and co-workers have developed a statistical theory,^57,58^ referred to here as SKV theory, which accurately predicts adsorption, desorption, and scanning branches of isotherms in statistically disordered chains of serially connected cylindrical long pores [statistically disordered chain model (SDCM)]. This is the simplest form of pore networks and, hence, is qualitatively different from IPM. It has been shown to capture the majority of the basic features of adsorption/desorption hysteresis encountered for fluids in mesoporous materials,^33,34^ as well as their scanning behavior.^59,60^ Employing mean field theory (MFT) for the lattice fluid model, Kikkinides et al.^61^ have demonstrated that SKV theory reconstructs very accurately all branches of the sorption isotherm for disordered pore chains. Even though this theory applied to SDCM has been extremely useful for obtaining insight into the complex behavior of phase transitions in disordered pore networks,^62,63^ its major bottleneck for the analysis of real porous solids is related to its very specific topology. This brings up the need for the development of a statistical theory for pore networks that can provide more realistic representations of the pore structure and can better capture percolation-like processes.

Recently, Kikkinides and Valiullin^64^ developed a new statistical theory that describes adsorption and desorption in mesoporous materials represented by pore networks in the form of Bethe lattices. The new theory is, in its spirit, an extension of that earlier applied to SDCM. It treats the structures with pore connectivity larger than two and incorporates the cooperativity effects both during the desorption and adsorption. The theory was validated against simulations and algorithmic models that describe sorption of lattice and real fluids in Bethe lattices. It was demonstrated that the pore network coordination number, or pore connectivity, *z*, has a significant impact on two important processes observed in pore networks: pore-assisting condensation during adsorption and evaporation by percolation during desorption. The inclusion of pore connectivity in the earlier developed framework accounting for cooperativity effects is an important step in rendering the existing models to mimic fluid behavior in real materials more accurately. Hence, the new theory inherently contains all essential elements that may offer the extraction of more reliable PSD and interpore connectivity, utilizing simultaneously both the adsorption and desorption branches of the isotherm.

In the present work, we demonstrate the application of the new statistical theory for the extraction of the basic structural and topological properties of the pore network PSD and pore connectivity, *z*, of real porous solids by developing a robust methodology to solve the inverse problem. The method is first validated by employing adsorption–desorption isotherms, determined on pore networks in the form of Bethe lattices of known PSD and *z*, with the use of a standard algorithmic model.^64^ Accordingly, the method is applied to extract PSD and *z* from N_2_ adsorption–desorption isotherms measured experimentally on Vycor porous glass, a porous material extensively by numerous experimental techniques. The resulting geometrical and topological properties are compared against the respective ones assessed from 3D images of stochastic reconstructions of this material, showing very good agreement between the two methods. Evidently, the current methodology that employs the recent statistical theory for pore networks, in the form of Bethe lattices, appears to be a promising tool for accurate and reliable pore structural characterization tool using gas physisorption experiments.

## 2 Methods

**Figure 1:**
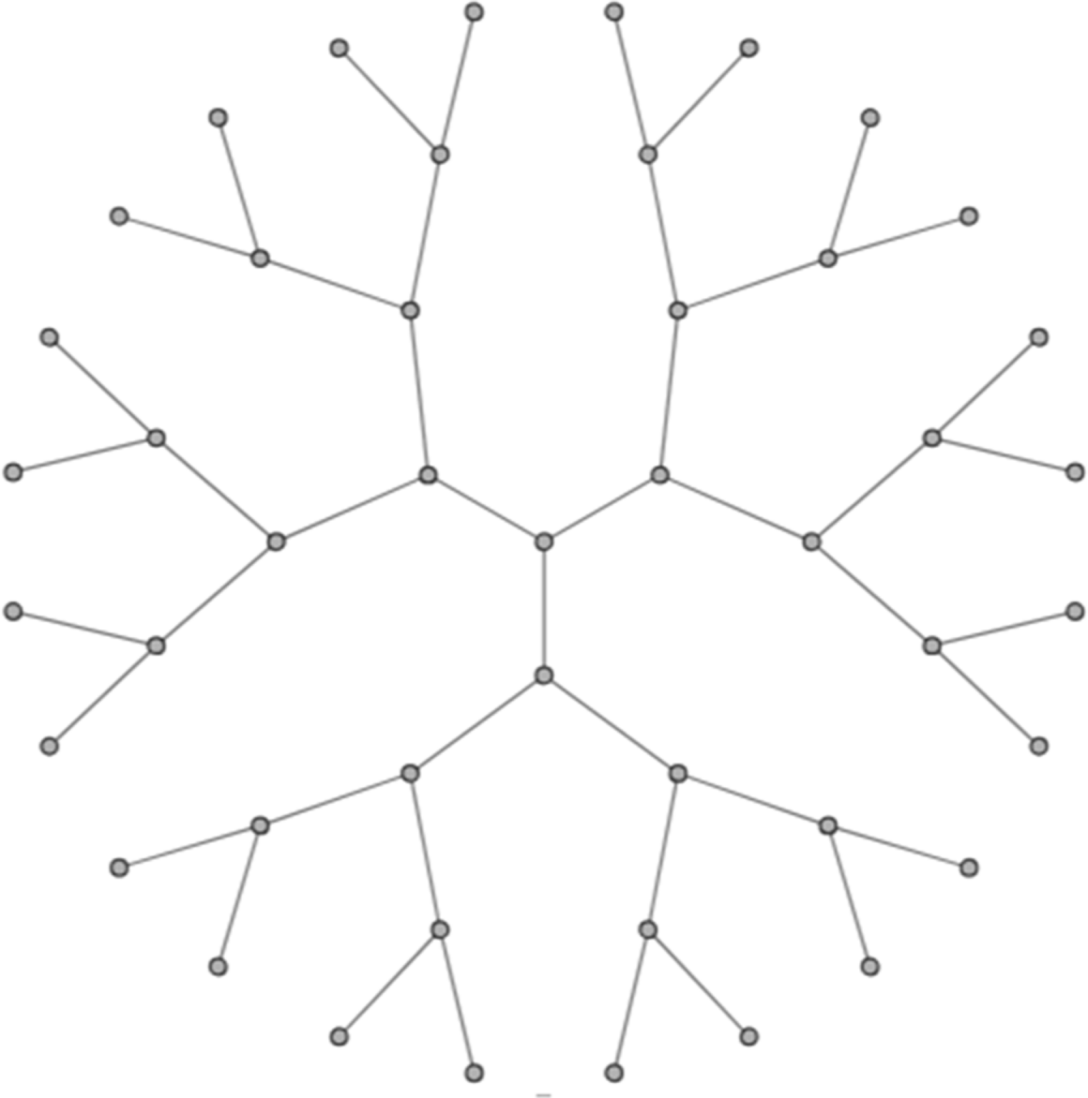
Schematic sketch of a Bethe lattice (*z* = 3).

In the present study, the Bethe lattice, also known as the Cayley tree, which is a Bethe lattice of finite size,^65^ is employed to represent the porous structure. It is shown schematically in Figure 1 and represents a loop-less lattice in which any two pores are connected by a single, unique pathway. In contrast to earlier dual site-bond models,^55,66^ we treat the junctions (sites) as volume-less, representing mere connections of *z* pores (bonds). Following refs (57,58, and 61), we consider two mechanisms for capillary-condensation and evaporation transitions in a pore, referred to as nucleation and phase growth. Their essence can most instructively be demonstrated using linear pore chains. In particular, condensation can occur by nucleation, which is liquid bridging in a pore in contact with vapor at both ends. This process is associated with the overcoming of the barrier in free energy and hence needs thermal activation. On the other hand, for a pore with one end closed by a condensed phase, it fills with the capillary-condensate by liquid invasion, i.e., by phase growth, and not by liquid bridging. Similar concepts apply for the desorption transition, in which nucleation is associated with cavitation in the limit of mechanical stability of the capillary-condensed phase, and phase growth is gas invasion either from bulk or from the pores in which cavitation has already occurred. How these two mechanisms are implemented for the Bethe lattice will be discussed later.

The conditions for nucleation and phase growth are encoded in the respective transition kernels for individual pores. The two nucleation kernels are denoted by θ_n_(*x*,*p*) for liquid bridging and θ_c_(*x*,*p*) for cavitation. Because of reversibility in thermodynamic equilibrium, the kernels describing the phase growth processes, namely, advanced condensation and gas invasion, coincide and can be described by the same kernel, θ_g_(*x*,*p*). The critical pore sizes, i.e., the pore sizes in which phase transitions occur at given vapor pressure by the mechanisms discussed, are denoted with *x*_n_(*p*), *x*_g_(*p*), and *x*_c_(*p*) for liquid bridging, advanced condensation/gas invasion, and cavitation, respectively.

### 2.1 Statistical Theory for Adsorption–Desorption in Pore Networks

It has been shown^58^ that, quite generally, the adsorption isotherm can be expressed as
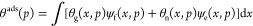
1where ψ_f_(*x*,*p*) and ψ_e_(*x*,*p*) are the volumetric PSDs of the filled (filled with the capillary-condensed liquid) and empty (containing the adsorbed film only) pore sections in the pore network, at a certain pressure, *p*. By definition, ψ_f_(*x*,*p*) + ψ_e_(*x*,*p*) = φ(*x*), where φ(*x*) is the volumetric PSD of the structure. This expression is valid for any topology of the pore space, including IPM too. Because eq 1 is constructed by explicitly using the functions ψ_f_(*x*,*p*) and ψ_e_(*x*,*p*), it automatically includes any network effects, which are encoded in these functions by an appropriate theory. Similarly, the desorption isotherm is determined as
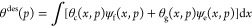
2

It is important to remind here once again that the fluid behavior at the pore junction (site) determines decisively the functions ψ_f_(*x*,*p*) and ψ_e_(*x*,*p*), hence, the overall system behavior.^67^ Note that, in SDCM, the junctions are just the connections between two adjacent pores, while in the Bethe lattice, more than two pores are connected to each other. We model the physics of how junctions affect the filling/emptying processes as follows: During adsorption, an open pore can be filled with capillary-condensate via advanced sorption,^45,55,56,64^ i.e., phase growth, only if the remaining (*z* – 1) pores (bonds) connected to the pore junction are filled with condensate. On the other hand, during desorption, if at least one pore connected to a pore junction evaporates, then all remaining pores connected to it can evaporate by gas invasion and not by nucleation (cavitation).^51,53,64^ More details on these processes and their validation employing MFT simulations and algorithmic models can be found in ref (64).

The concept just outlined is constructed by neglecting the volume of the junctions (sites) connecting the bonds in the lattice. This provides a simpler description of the pore geometry and does not require the introduction of a separate PSD for the junctions. In principle, one may consider that these junctions have a size that is larger or equal to the connecting bonds (the construction principle, CP).^55,66^ This means that during desorption and in the absence of cavitation, each site cannot evaporate unless one of the connecting bonds evaporates first. Hence, the size of the site does not affect the mechanism of desorption due to pore blocking. However, during adsorption, the size of the site matters, since the assisting condensation process for pore filling requires that (i) all (*z* – 1) pores connected in this site must be filled with condensate and (ii) the connecting site must also be filled with condensate.^64^ Condition (ii) can be accomplished if the size of junction is comparable to the sizes of the intersecting pores, or, simply, a pore extension or buffer zone where neighboring pores meet.^64^

### 2.2 Construction of the Adsorption Branch

During a quasi-static rise of gas pressure, the pore sections fill gradually. Since *x*_g_(*p*) > *x*_n_(*p*) for any pressure *p*, the PSD of the filled sections, ψ_f_(*x*,*p*), can be shown to be a piecewise function^58^
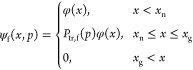
3

Equation 3 implies that filling of every pore section with a size *x* ≤ *x*_n_ is triggered by nucleation. For *x*_n_ < *x* ≤ *x*_g_, a pressure-dependent function *P*_tr,f_(*p*) determines the fraction of the pore sections in which the liquid phase can be formed due to phase growth from the pore sections in which the liquid phase was already formed by nucleation, i.e., due to advanced sorption (assisting process). Finally, pore sections with size *x* > *x*_g_ remain empty because they cannot be filled by either mechanism. Substituting eq 3 into 1 the latter becomes
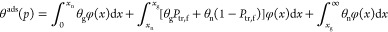
4or in a compact form
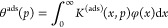
5where *K*^(ads)^(*x*,*p*) is a generalized adsorption kernel depending on both, pressure, *p*, and, pore size, *x*.

*P*_tr,f_ can be determined by using the mean probabilities *P*_n,f_ and *P*_g,f_ that an arbitrarily selected pore section has a sufficiently small size to allow filling by nucleation or phase growth, respectively. They are equal to the respective cumulative probabilities  of the normalized number distribution functions *h*(*x*) = φ(*x*)/*x*^2^ for cylindrical pores.^48^ Thus
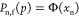
6and
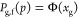
7

According to SKV theory, a pore fills with condensate during adsorption by either nucleation or growth.^58^ In our recent work,^64^ we have shown that, for a pore network with coordination number *z*, it holds
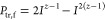
8where *I* is the probability of a neighboring bond to fill with condensate, which is related to *P*_n,f_ and *P*_g,f_ by the following expression
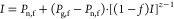
9

In eq 9, *f* is a phenomenological parameter accounting for the finite-size effects and representing the fraction of pores in the Bethe lattice having contact to the external gas phase. Equation 9 is a nonlinear algebraic equation that is solved iteratively to determine *I* for various values of *z*. Hence, the adsorption branch is constructed by eq 4 with the aid of eqs 8 and 9. Note that for the special case of a Bethe lattice with *z* = 2, we recover the expression for *P*_tr,f_, which has been derived independently for long chains of SDCM structures.^58,64^

### 2.3 Construction of the Desorption Branch

The analysis for the desorption branch of the isotherm is performed in the same way. In this case, the critical pore diameters for nucleation (cavitation) and growth (gas invasion) are *x*_c_(*p*) and *x*_g_(*p*), respectively. Upon a quasistatic decrease of the gas pressure, the pore sections empty gradually, and the PSD of the empty pore sections is determined as follows
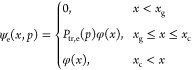
10

Substituting eq 10 into 2 the latter becomes
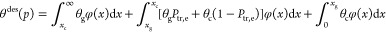
11which once again can be written in a compact form as
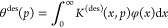
12where also in this case, *K*^(des)^(*x*,*p*) is the generalized desorption kernel depending on both, pressure, *p*, and, pore size, *x*.

In the same line with the adsorption case, *P*_tr,e_ in eq 10 is established by using the mean probabilities for cavitation, *P*_n,e_(*p*), and gas invasion, *P*_g,e_(*p*)
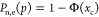
13and
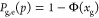
14

For a Bethe lattice, *P*_tr,e_ is given by^53^
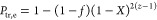
15where *t*(*z*) = 2(*z* – 1) is the perimeter of a bond (pore) in the Bethe lattice,^65,68^ while *X* is the probability that a neighbor to the pore provides connection to the vapor phase. The latter probability is^50,51,53^
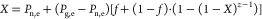
16

Equations 15 and 16 can be used to determine *P*_tr,e_ (*p*), and, thus, the desorption transition as given by eq 12. Note that, for an infinite Bethe lattice (*f* = 0) with *z* = 2, we recover the expression for *P*_tr,e_ that has been derived independently along the course of SKV theory for SDCM structures with infinitely long chains.^58,61,64^ The other limit at *z* → ∞, gives *P*_tr,e_ = 1, which is the IPM solution for desorption.^58,64^

### 2.4 Extraction of PSD from Isotherm Data Using SKV Theory

It is well-known that the problem of determining the PSD from a given isotherm is, in general, an ill-posed one. The adsorption–desorption integral equations, eqs 4 and 12, are the Fredholm equations of the first kind, and the inversion of such equations is a challenging problem, manifested by the fact that even small changes in the experimental data may cause large changes in the final solutions.^27^ Hence, several stabilization methods have been employed to obtain meaningful results in terms of the extracted PSD. The best-known method for stabilization is the so-called regularization method introduced by Tikhonov (for a review on the subject, see ref (27)). In all these applications of the regularization method, the PSD function φ(*x*) is discretized and evaluated at a certain number of discrete pore sizes, called nodes. Hence, we must first set the limits of integration from [0, ∞] to a finite region of [*x*_min_, *x*_max_], with *x*_min_ and *x*_max_ being the respective lower and upper pore sizes of the PSD that we want to extract. Evidently, these values are not known a priori, and a trial-and-error procedure is needed to improve the accuracy and resolution of the extracted PSD.

Since we seek to extract a discrete PSD in [*x*_min_, *x*_max_], we first replace integrals by sums in eqs 5 and 12, which are then written as follows
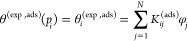
17and
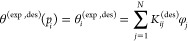
18

The above equations can be written for a set of *M*_a_ pressure points for adsorption, and *M*_d_ pressure points for desorption, with a total number of pressure points, *M* = *M*_a_ + *M*_d_. While *N* is the number of discrete cylinder sizes for which we have input kernels. Thus, we employ the following general set of equations, for *i* = 1...*M* and *j* = 1...*N* (*N* < *M*)
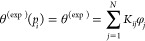
19with
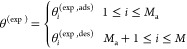
20and
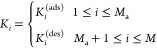
21

The kernels *K*_*ij*_^(ads)^ and *K*_*ij*_^(des)^, are determined by complex expressions that depend on relative pressure, *p*/*p*_0_, as well as structural properties, including, the unknown PSD **φ**, at the discrete points *x*_*j*_ (*j* = 1...*N*), pore connectivity, *z*, and finite size parameter, *f*. These expressions are given in detail in the Supporting Information section.

Accordingly, we employ an iterating scheme to determine *f*, *z*, and **φ**, minimizing the following general nonlinear least-squares problem
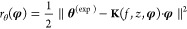
22

Details on the methodology developed and employed to solve the above problem are given in the Supporting Information section. Note that for most practical applications of real mesoporous materials, which are not very fine powders, the finite size parameter, *f*, which gets values in [0, 1], is usually quite small, *f* < 0.01. For such small values of *f*, its variation leads to substantially smaller changes in the final solution compared to those of *z* and φ.

### 2.5 Solution Stabilization and Smoothing Using Cubic B-Splines

A major drawback of the methodology described in the previous section is that because it is based on direct discretization of integral adsorption–desorption equations, the number of nodes in pore sizes, *N*, must be smaller than the number of points in pressure, *M*, to get a solution.^69^ Moreover, it must hold *N* ≪ *M* to get a stable and meaningful PSD solution.^69,70^ This significantly limits the resolution of the PSD and hence the accuracy of the solution since several terms in eqs 5 and 12, contain integrals that depend on the resolution in pore size.

The best way to resolve this issue is by considering the solution φ(*x*) represented by piecewise continuous functions, such as cubic B-splines.^69,70^ The advantage of employing this procedure is that one can use a very fine resolution in terms of a chosen number of pore sizes, *N*_*x*_, ensuring a high accuracy in the solution of the various integrals in eqs 5 and 12, while solving the problems for a small number of B-spline nodes,^69,70^*N*, so that *N* ≪ *M* (but *N*_*x*_ > *M*). Further details, showing quantitatively how cubic B-splines are employed in the present study, are given in the Supporting Information section.

## 3 Results and Discussion

### 3.1 Validation of the Proposed Methodology for Bethe Lattices

Before applying the above outlined theory to real experimental isotherms, we apply SKV theory to extract volume PSDs from adsorption–desorption isotherms obtained by the algorithmic model on Bethe lattices^64^ made from cylindrical pores with a predefined PSD, connectivity value, *z*, and finite size parameter, *f*. This will allow us to prove that the method we have developed to solve the inverse problem is correct. Hence, we validate the above methodology employing as “experimental” isotherms the ones obtained by the algorithmic model for Bethe lattices^64^ of a preset pore connectivity, *z* = 3, finite size parameter, *f* = 0.01, and PSD, φ(*x*). In the algorithmic model, a random network is generated, and phase transitions in this resulting structure are traced by considering local geometric order according to the predefined transition rules. A large number of random network realizations have been considered (typically between 10^3^ and 10^4^). Note that it was recently shown that the SKV theory for Bethe lattices is in excellent agreement with the algorithmic model.^64^

For the sake of simplicity, the capillary model for sorption behavior is used for the analysis, assuming cylindrical pores and employing the Kelvin–Cohan model for pore condensation–evaporation of N_2_ at 77.4 K, along with the Harkins–Jura equation for the statistical thickness of the adsorbed layer, which has been shown to be quite accurate for pore sizes above 10 nm.^11,13^ Note that we choose the capillary model to be complement with our earlier work on the validation of SKV theory for Bethe lattices with the respective algorithmic model.^64^ Using more advanced models will have only quantitative effects and will not change the conclusions made using the capillary model. Following our recent work,^64^ we consider normal (Gaussian) number PSDs with average size, *x̅* = 10 nm, and standard deviations, σ = 2 nm. We employ our methodology with *x*_min_ = 2 nm, *x*_max_ = 20 nm, and with number of B-spline nodes, *N* = 11, while *N*_*x*_ = 201. A finer discretization has also been employed; however, no notable change in the results has been noted. For the sake of simplicity, we have skipped the need for optimizing the finite size parameter, *f*, by fixing it to *f* = 0.01. We have already demonstrated^64^ that, for large pore networks with *f* ≪ 1, the effect of varying *f* is marginal.

**Figure 2:**
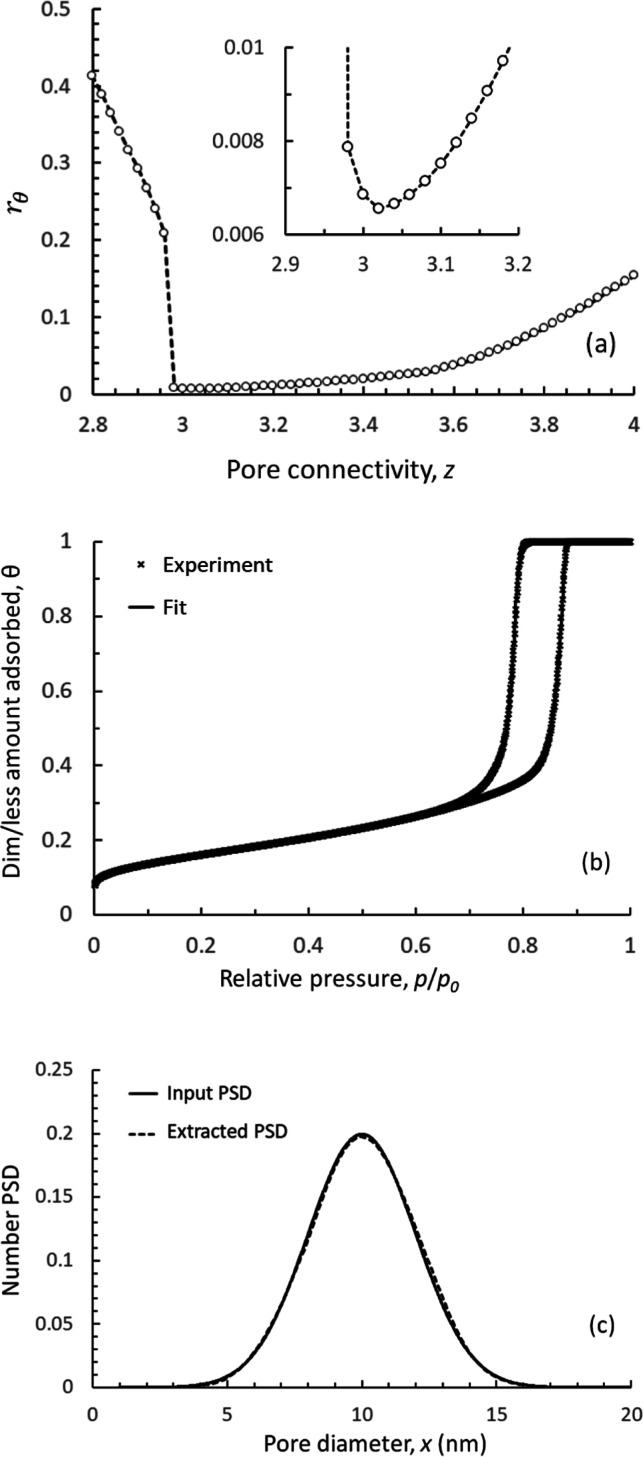
(a) Convergence of the objective function, *r*_θ_, with respect to *z*, for a sorption isotherm produced by the algorithmic capillary model in a Bethe lattice with *z* = 3, with pore sizes obtained from a Gaussian number distribution with *x̅* = 10 nm, and σ = 2 nm. Detail in the vicinity of the convergence region is shown in the indent. (b) Comparison between input and fitted adsorption–desorption isotherms, and (c) original and extracted PSD.

In Figure 2a, we illustrate the iterative procedure in *z*. The optimal solution is obtained for *z* = 3.02, which is in excellent agreement with the preset value of *z* = 3 (relative error 7 × 10^–3^). In Figure 2b, we compare the “experimental” and predicted isotherms. It is seen that there is an excellent matching between the two. Furthermore, in Figure 2c, we compare the predefined Gaussian number PSD with the one extracted by the above methodology, showing, once again, an excellent agreement between them. It must be noted that we have employed a small number of B-spline nodes in the fitting process, *N* = 11, compared to the total number of pressure points, *M*, for both branches of the isotherms (*M* = 2002), for *x*_min_ = 2 nm and *x*_max_ = 20 nm, while keeping a high resolution in the PSD analysis with *N*_*x*_ = 201.

Additional validation studies are employed, considering different values of *z* or *f* showing similar results with those presented above. Hence, we conclude that the proposed methodology can be employed to extract reliable structural information and, more importantly, both PSD and pore connectivity, *z*, of mesoporous networks based on adsorption–desorption isotherms.

### 3.2 Extraction of Structural Information on Real Porous Materials

After validating the methodology for solving the inverse problem, we demonstrate its application to extract PSD and pore connectivity, *z*, of real porous materials using standard N_2_ sorption isotherms at *T* = 77.4 K. We choose to apply our methodology to the case of Vycor porous glass (7930), as this material has been commonly used as a model porous structure with significant disorder effects and has been extensively studied by different computational^31,71^ and experimental techniques.^72^ Furthermore, there exist stochastic reconstruction methods that have been successfully employed in the past to produce accurate 3D digitized binary replicas of the Vycor pore structure that preserve the basic structural properties of the material, measured directly by TEM and indirectly by small angle X-ray or neutron scattering techniques (SAXS or SANS, respectively).^73,74^ The produced 3D digital images can be further employed to extract geometric PSD and pore connectivity with the use of advanced computational characterization techniques that exist in the open literature,^32,75,76^ and these findings can be used to corroborate the results of the present study.

### 3.3 Transition Kernels

An important issue in extracting reliable structural information from adsorption–desorption isotherms is the use of accurate sorption kernels of different gases on pores of a certain geometry and size. To this end, there has been quantitative information from NLDFT and GCMC simulations of N_2_ and Ar on cylindrical silica (SiO_2_) pores, while several semimacroscopic models based on the capillary model have been proposed.^14,16,21–24,77^ Among these models, the one proposed by Bonnet–Wolf (BW model^22^) and later modified by Morishige^24^ appears to provide quite accurate kernels for N_2_ and Ar sorption on cylindrical SiO_2_ pores. These authors have considered thermal activation on the condensation and evaporation of a fluid confined in a cylindrical pore based on the grand potential of a bubble in the pore core. For the case of N_2_, thermal activation can lead to condensation pressures that are distinctly lower than the vapor spinodal for pore sizes below ∼10 nm and cavitation during evaporation, while for pore sizes below 5 nm, the thermal condensation branches coincide with the equilibrium evaporation branch. These findings are in reasonable agreement with experimental results obtained in ordered MCM porous materials of defined shapes and sizes that have been determined by direct methods.^23,24^

When comparing the work of BW^22^ with that of Morishige,^24^ it is seen that the major difference between the two is the solid–fluid interaction potential employed. BW have considered the attractive part of the cylindrical Lenard Jones 9-3 (CLJ 9-3) potential,^22^ in accordance with Saam and Cole’s theory.^77^ This interaction potential considers pore walls of infinite thickness and predicts a shallow minimum located too close to the cylindrical surface. Morishige,^23,24^ on the other hand, employs the CLJ 10-4 potential developed by Tjatjopoulos et al.,^78^ which considers pore walls of zero thickness.^79^ This potential has been previously employed in the adsorption analysis of MCM-41 types of materials and single-wall carbon nanotubes, and in general, it works well for pores of very small pore-wall thickness. A third model, considered in the present work, is the CLJ 10-4-3 potential developed by Siderius and Gelb,^79^ which, in the limit of large radii coincides with the celebrated Steele’s 10-4-3 potential for planar surfaces.^80^ Although Steele’s 10-4-3 potential is generally applied only for graphitic structures, the form of the potential was also suggested to be an appropriate representation of fluid–solid interactions involving fcc lattices with exposed (111) and (100) surfaces.^81^ Evidently, CLJ 10-4-3 corresponds to an approximate representation of a multilayered cylindrical pore, where the first layer of atoms is represented by a wall of zero thickness and uniform surface density, and the rest of the solid is integrated starting from a distance αΔ from the surface, representing a cylinder of uniform volumetric density, keeping only the attractive part of the pairwise potential of the fluid–solid interaction.^81^ Parameter α was set to α = 0.61 by Steele for the case of graphite, but it also has some theoretical grounds^82^ that justify its use for any type of material. The CLJ 10-4-3 potential is always more strongly adsorbing, exhibits a deeper minimum than the CLJ 10-4 potential, and is expected to work better for pores with walls of finite thickness. Note that for the case of silica, the value of thickness Δ, is set to Δ = 0.36 nm.^83,84^ Further details on the CLJ potentials are provided in the Supporting Information section.

**Figure 3:**
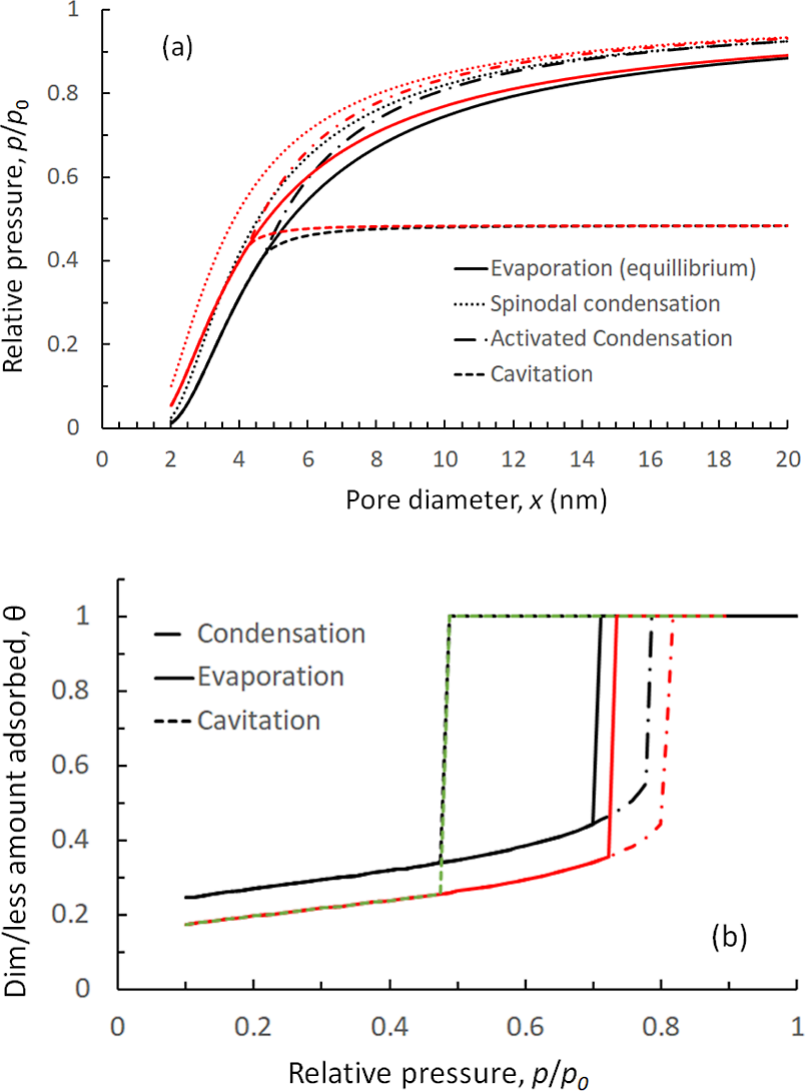
(a) Relations between relative pressure and pore diameter, for N_2_ phase transitions at *T* = 77.4 K, during evaporation (equilibrium), spinodal and activated condensation, and cavitation. (b) N_2_ sorption Kernels at 77.4 K, for activated condensation, evaporation (equilibrium), and cavitation in a cylindrical pore of 9 nm diameter. BW theory is employed by CLJ 10-4-3 (black lines) and CLJ 10-4 (red lines) potentials, to model solid–fluid interactions.

In Figure 3a, we present the relation between pore diameter and relative pressures of spinodal and activated condensation, equilibrium evaporation, and cavitation, employing CLJ 10-4-3 and CLJ 10-4 potentials for solid fluid interactions. The results based on CLJ 10-4-3 potential, are in excellent agreement, for pore diameters above ∼3 nm, with the recently reported data by Söllner et al. (2024),^85^ which are based on NLDFT for condensation, equilibrium evaporation, and^26,27^ molecular simulations for cavitation,^86^ combined with experimental data on model materials to account for the correct phase behavior, particularly for pore sizes below 5 nm.^85^ On the other hand, the CLJ 10-4 potential predicts nucleation and growth transitions at higher relative pressures compared to CLJ 10-4-3. Figure 3b exemplifies the transitions associated with nucleation, growth (equilibrium), and cavitation for a cylindrical pore with diameter *d*_p_ = 9 nm. It is seen that all transitions predicted when using the CLJ-10-4-3 potential are once again in excellent agreement with the respective results by Söllner et al. (2024),^85^ while the use of the CLJ 10-4 potential predicts higher relative pressures for nucleation and growth transitions, in accordance with the results presented in Figure 7a.

**Figure 4:**
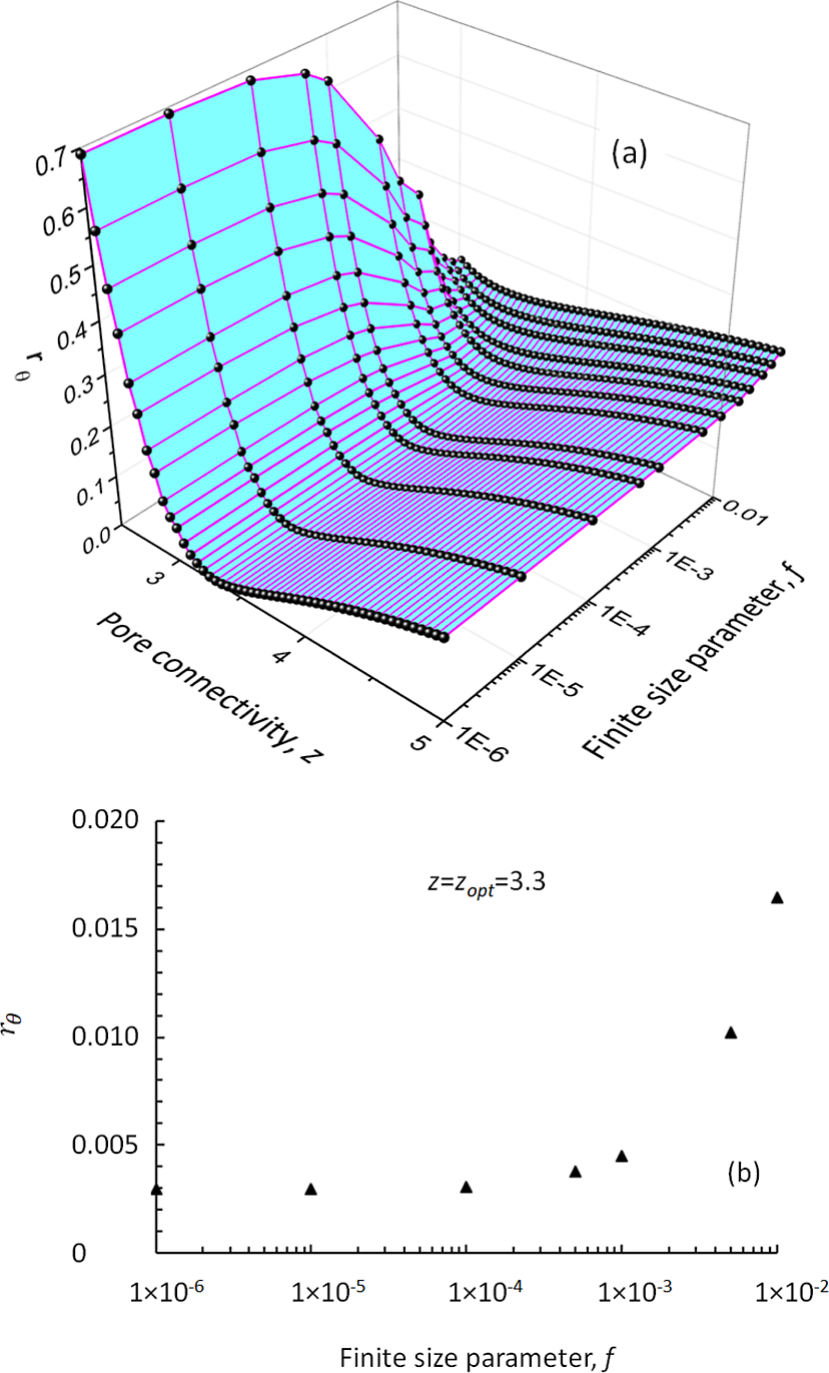
(a) Convergence of the objective function, *r*_θ_, with respect to *z*, and *f*, for a N_2_ sorption isotherm on Vycor porous glass at *T* = 77.4 K, employing SKV theory for Bethe lattices and (b) effect of a finite size parameter, *f*, on *r*_θ_ at the optimal value of *z* = 3.3.

**Figure 5:**
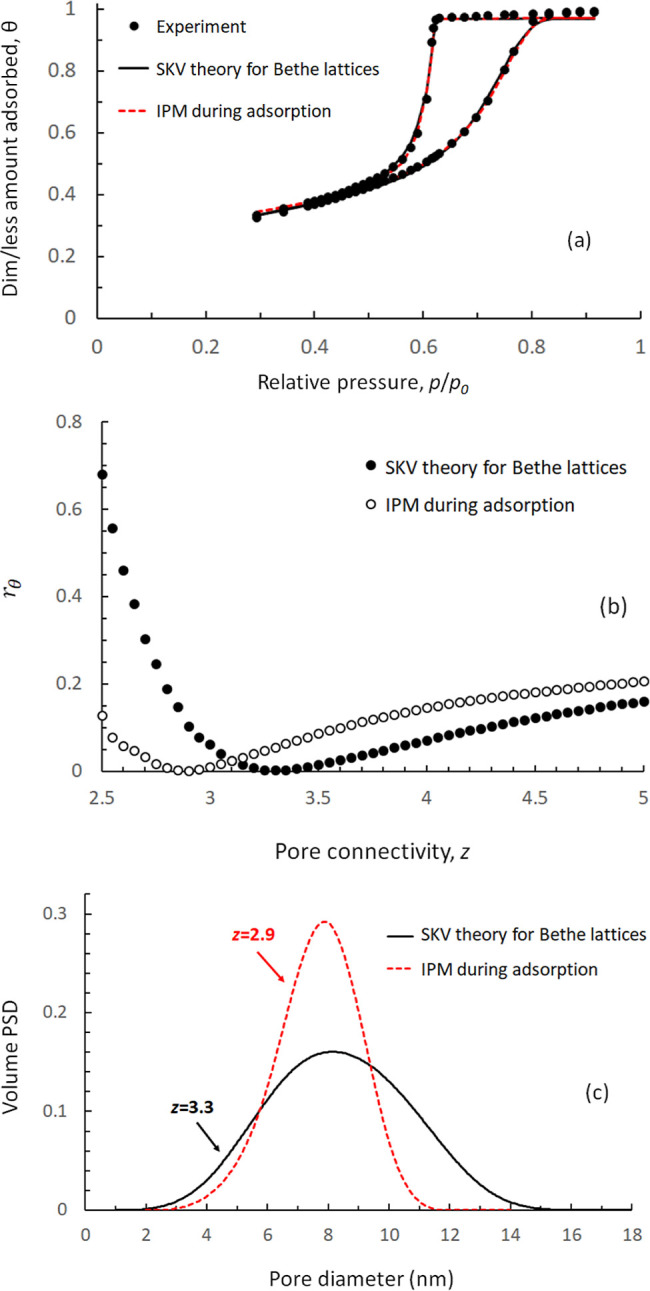
(a) Comparison between experimental and fitted adsorption–desorption isotherms for N_2_ on Vycor porous glass, at *T* = 77.4 K, employing the present method based on SKV theory for Bethe lattices, and a variation of the method, where IPM is considered for the adsorption branch and percolation for the desorption branch. (b) Iteration procedure to obtain optimal *z* and (c) extracted PSDs at optimal *z*, from N_2_ sorption data, employing each method (*f* = 1 × 10^–4^).

**Figure 6:**
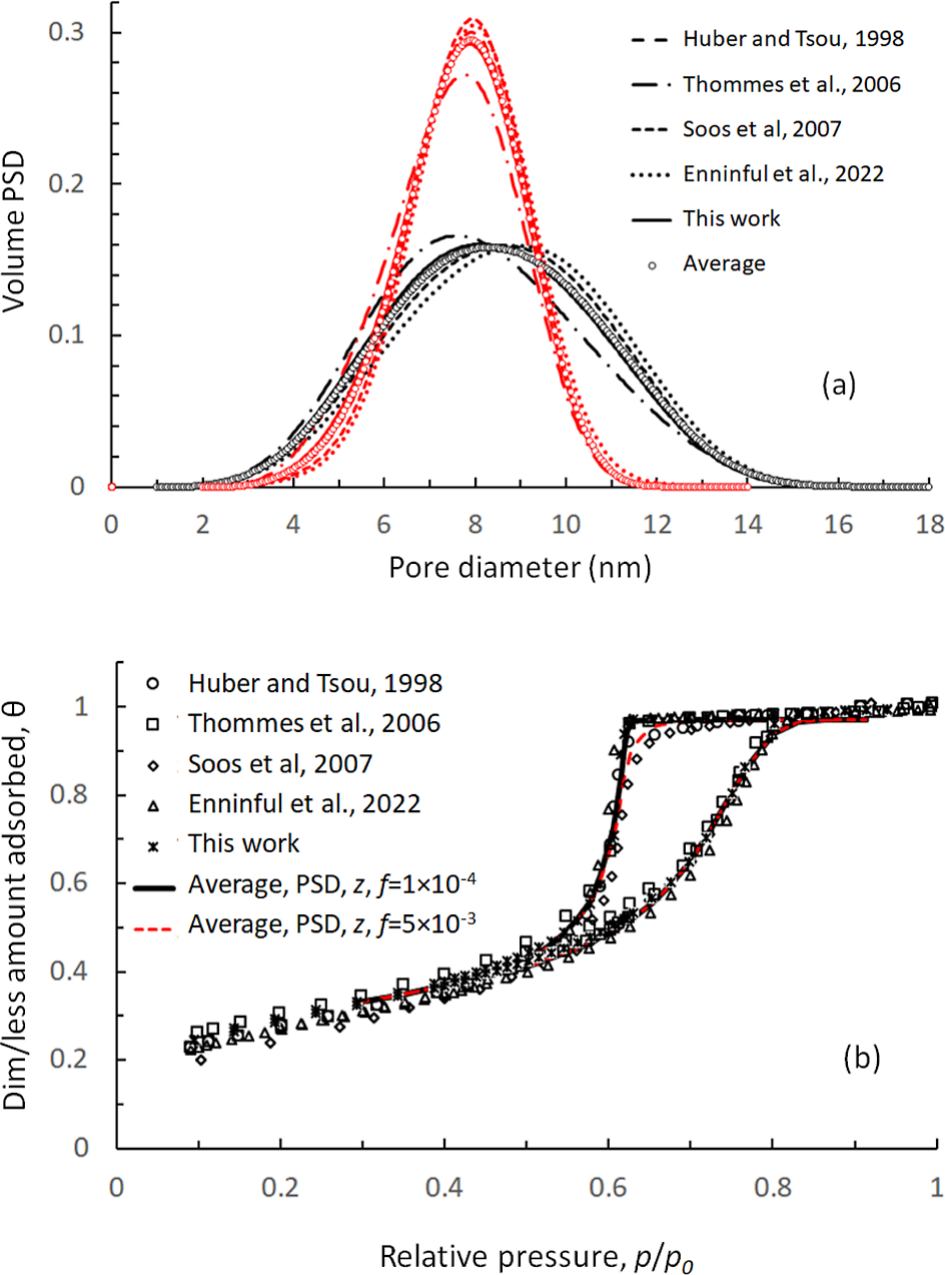
(a) Extracted volume PSDs from N_2_ (77.4 K) adsorption–desorption isotherms on Vycor porous glass, measured by different research groups, employing the present method based on SKV theory for Bethe lattices, and a variation of the method, where IPM is considered for the adsorption and percolation for the desorption branch (in red). (b) Comparison between a theoretical N_2_ sorption isotherm, predicted from SKV theory for Bethe lattices using average pore structural values and experimental N_2_ sorption isotherms measured by different research groups (*T* = 77.4 K).

**Figure 7:**
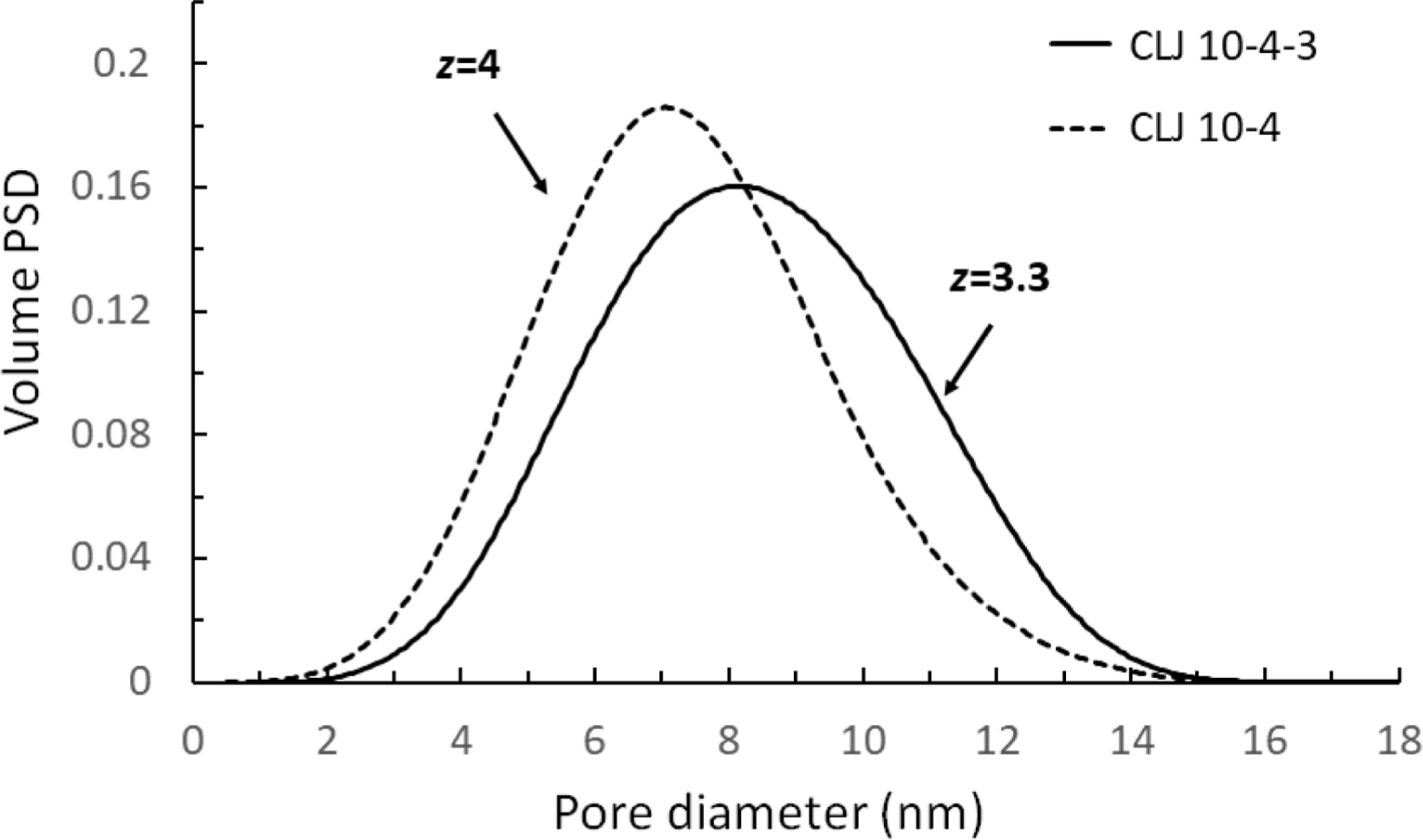
Effect of the solid–fluid interaction potential employed in the BW model used in the present method to extract PSD and *z* from N_2_ (*T* = 77.4 K) adsorption–desorption isotherms.

Hence, the employment of CLJ-10-4-3 potential to model solid–fluid interaction in BW theory, provides quite accurate kernels that can be further employed in our statistical theory to extract structural data for real mesoporous materials. On the other hand, the use of CLJ 10-4 potential is expected to provide PSDs with a small bias toward smaller pore sizes compared to the CLJ 10-4-3 potential.

### 3.4 Analysis of Sorption Isotherms for Vycor Porous Glass

With the accurate kernels available for sorption data in cylindrical pores, we can apply our methodology to extract structural information on Vycor porous glass. For the experiments, we have used commercially available Vycor glass. The original monolith was crashed into millimeter-large pieces to reduce the equilibration times, controlled by slow N_2_ diffusion at low temperatures. The sample was degassed at 523 K for 10 h under ultrahigh vacuum. The nitrogen sorption measurements were performed at 77.4 K in the relative pressure range *p*/*p*_0_ from the lowest possible to 0.995 with a Quantachrome autosorb iQ (Quantachrome Instruments, Anton Paar QuantaTec). For the adsorption isotherm, the mesopore range was investigated in detail with very small intervals of 0.025*p*/*p*_0_. In the case of the desorption isotherm, 0.0125*p*/*p*_0_ intervals were used between *p*/*p*_0_ 0.995 and 0.1. For each data point, an equilibration time of 6 min was set. To test the robustness of the present methodology, we have also used N_2_ adsorption–desorption experimental isotherm data on Vycor measured by several other research groups.

Since our kernels for N_2_ are in excellent agreement with NLDFT for pore diameters above ∼3 nm, and thus for relative pressures, *p*/*p*_0_ ≥ 0.3, we chose to fit the isotherms beyond this value. In the minimization technique described above, we have employed *x*_min_ = 1 nm, *x*_max_ = 19 nm, *N* = 7, and *N*_*x*_ = 301, although we have considered finer resolution (*N*_*x*_ = 3001) with no significant effect in the results. In addition to the employed SKV theory for Bethe lattices, we have developed a variation of the theory, where IPM is employed to the adsorption branch only, while percolation theory for Bethe lattices is employed in the desorption branch. This modification is in full accordance with previous studies of Seaton and co-workers^87,88^ (for random pore networks) and Pinson et al.^53^ (for Bethe lattices). In this case, we have employed, after some trial and error, *x*_min_ = 2 nm, *x*_max_ = 14 nm, *N* = 7, and *N*_*x*_ = 201. Note that the total number of pressure points ranged from *M* = 42 to *M* = 60, depending on the isotherm dataset used. In all cases, *M* ≫ *N* ensuring stable results in the extracted PSD.

In Figure 4, we illustrate the procedure employed to extract the optimum value of pore connectivity, *z*, and the finite size parameter,*f*, using SKV theory for Bethe lattices. More specifically, in Figure 4a, we present a 3D plot with the variation of *r*_θ_ with *z* and *f*. It is found that the optimal value of *z*, that minimizes *r*_θ_, is *z* = 3.3, while *f* has practically no effect on *r*_θ_ for *f* ≤ 1 × 10^–4^. This is better seen in Figure 4b, where *r*_θ_ is plotted against *f*, at the optimal value of *z* = 3.3.

Hence, we can conclude that a set of values *f* = 1 × 10^–4^ and *z* = 3.3 produce an optimal PSD that gives the best fit of both N_2_ adsorption and desorption isotherms on Vycor porous glass.

An important aspect of the present study is to compare the new theory with previous approaches in terms of the extracted structural properties from sorption isotherms. Hence, in Figure 5a, we compare experimental and fitted isotherms for N_2_ on Vycor porous glass, employing SKV theory for Bethe lattices and a variation of this method, where IPM is employed in the adsorption branch only, while percolation theory for Bethe lattices is employed in the desorption branch. To recall, the latter (hybrid) model is essentially used earlier by Seaton and co-workers,^87,88^ for random pore networks, and by Pinson et al.,^53^ for Bethe lattices. It is seen that there is an excellent fit of experimental adsorption–desorption isotherms on Vycor, using either fitting model. Note that in the present study, we have chosen to ignore the compressibility effects of the liquid density of the adsorbates for the sake of simplicity, and in accordance with the basic assumptions in the theory for activated condensation–evaporation in the BW model.^47^ We expect that this simplification should have a limited effect on the extracted structural information, and we plan to include them in future studies.

Since both methods provide an excellent fit of adsorption–desorption isotherms, we proceed with the comparison of the extracted structural information using either method. In Figure 5b, we present the iteration procedure to extract optimal values for *z* employing SKV theory for Bethe lattices and the hybrid model, combining IPM during adsorption with percolation during desorption. The finite size parameter,*f*, has been set to *f* = 1 × 10^–4^, in accordance with the conclusions drawn from Figure 4a,b, and by using the fact that Vycor porous glass was applied in the present study as essentially monolith. It is seen that the extracted optimal value of pore connectivity of Vycor porous glass drops from *z* = 3.3 (SKV theory) to *z* = 2.9, when IPM with neglected connectivity effects is employed in the adsorption branch and these effects are considered only in the desorption branch. In Figure 5c, we present the extracted volume PSDs for N_2_ sorption data employing the two approaches. The PSD produced by SKV theory is found to be essentially wider as compared to that produced by the hybrid model and shifted toward larger pore sizes, with the volume average pore diameter being ∼8.4 nm from SKV theory and ∼7.7 nm from the hybrid approach, employing IPM during adsorption.

As detailed experimental N_2_ adsorption–desorption isotherms on Vycor porous glass at 77.4 K have appeared in several publications over the past 30 years, this collection of independent experimental data can be used to test the robustness of our approach toward experimental scatter from different samples and measurements. Hence, besides our present data, we have considered isotherm data by Huber and Tsou (1998),^89^ Thommes et al. (2006),^90^ Soós et al. (2007),^91^ and Enninful et al. (2022).^92^ The extracted PSDs for each case, as well as the average PSD, are presented in Figure 6a. A small difference is observed among the various PSDs, which is quite reasonable, given the differences in samples and measurements. Similar differences are observed in the extracted values of *z* and *f* ,as summarized in Table 1.

**Table 1:** Pore Connectivity and Finite Size Parameter Values from Different Experimental N_2_ Sorption Isotherms on Vycor

research study	pore connectivity, *z*	finite size parameter, *f*
Huber and Tsou, 1998^89^	3.20	1 × 10^–^^3^
Thommes et al., 2006^90^	3.60	1 × 10^–^^4^
Šoóš et al., 2007^91^	3.25	5 × 10^–^^3^
Enninful et al., 2022^92^	2.90	1 × 10^–^^4^
this work	3.30	1 × 10^–^^4^
average	3.25	

Regarding the optimal value of the finite size parameter, *f*, it is seen that for the data from refs (90 and 92) and the present study, *f* = 1 × 10^–4^, which implies that *f* has a negligible effect on the pore structure of these Vycor samples. However, for the data of refs (89 and 91), higher values of *f* are needed to match the desorption branches. The latter, however, still fulfills the condition *f* ≪ 1.

In addition, in Figure 6b, we compare the N_2_ adsorption–desorption isotherm, constructed by the new theory employing averaged PSD and *z*, with the various experimental N_2_ sorption isotherms used to extract these average structural data. The agreement is remarkable, ensuring the uniqueness and robustness of the present methodology. Note that we have employed two different values of the finite size parameter *f*: *f* = 1 × 10^–4^ and *f* = 5 × 10^–3^. The latter value is used to match the desorption branches in the experiments reported in ref (91), but has no other effect in PSD and *z*.

### 3.5 Effect of Sorption Kernels

We have previously demonstrated the notable advantage of using CLJ 10-4-3 over CLJ 10-4 in getting much more accurate sorption kernels for N_2_ on silica (SiO_2_) cylindrical pores. Nevertheless, it would be interesting to explore the effect of using CLJ 10-4 instead of CLJ 10-4-3, on the extracted structural data from experimental N_2_ sorption isotherms on Vycor glass. Hence, in Figure 7, we compare the extracted PSDs and pore connectivity values from N_2_ sorption isotherms at 77.4 K on Vycor porous glass, measured in this work, using kernels for N_2_ on SiO_2_ pores, with CLJ 10-4-3 and with CLJ 10-4 solid–fluid interaction potential in the BW model. As expected, the use of CLJ 10-4 potential produces a volume PSD that is biased toward lower pore sizes. More specifically, the volume averaged pore size is ∼7.1 nm, employing CLJ 10-4, and ∼8.4 nm, employing CLJ 10-4-3, (relative difference ∼15.5%). This is because the respective kernels for nucleation and growth predict transitions at higher relative pressures compared to both BW models with CLJ 10-4-3 potential and NLDFT simulation data for pore sizes in the region 3–10 nm. The value of pore connectivity using CLJ 10-4 is *z* = 4, which is 21% higher than the respective value of *z* = 3.3, employing CLJ 10-4-3 potential. Hence, the employment of CLJ 10-4 potential for solid–fluid interactions, results in a PSD shifted to lower pores sizes and a higher pore connectivity, *z*, with relative differences of 15.5 and 21%, respectively.

### 3.6 Comparison with Structural Properties from 3D Stochastic Reconstruction

As a final step, we compare the present method that uses information from sorption isotherms to extract structural properties, with direct geometric methods that measure the same properties on 3D structures of the same porous materials. For the case of Vycor porous glass, there have been several 3D stochastic reconstruction methods that are known to produce quite accurate representations of the actual pore structure of this material in terms of its basic structural properties, such as, porosity, surface areas, two-point correlation function, pore, and mass chord-length distribution.^73,74,93^ These properties can be easily measured in the produced 3D digital images of reconstructed Vycor porous glass and have been measured directly or indirectly by advanced experimental techniques, including TEM, SAXS, and SANS.^72,74^

In the present study, we employ the model by Crossley et al. (1991),^73^ which has been proposed to generate 3D replicas of Vycor porous glass that preserve the structural and transport properties of the real material. This model has been applied in the past by one of us to generate 3D digital images of Vycor that have been shown to match porosity, two-point correlation function, surface area, and mass and pore chord length distributions that have been measured directly by TEM, and indirectly by SAXS and SANS.^74,93^ Furthermore, it was shown that the respective 3D images of Vycor, can be used as input digital domains to perform diffusion and viscous flow simulations and determine several transport properties, including molecular and Knudsen diffusion coefficients, viscous flow permeability, etc., that are in very good agreement with the respective transport properties measured by macroscopic experiments.^93,94^ Hence, we believe that 3D images generated by the model proposed by Crossley et al. (1991),^73^ provide realistic structural representations of Vycor porous glass and can be further used in the present work to extract additional structural parameters, such as PSD and pore connectivity, *z*.

**Figure 8:**
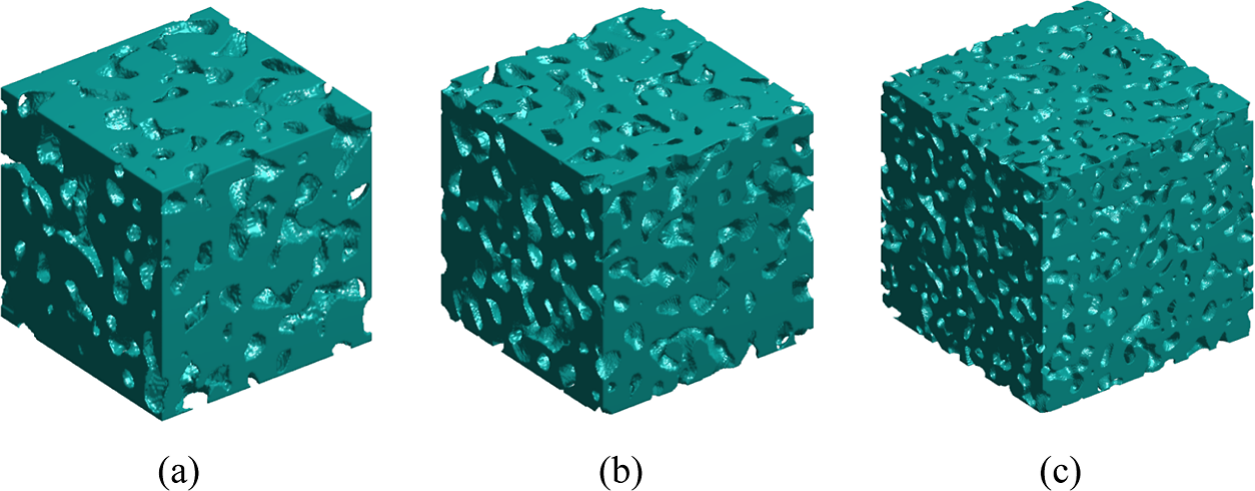
3D visualization of stochastic reconstructed digital images of Vycor porous glass with pixel size 0.75 nm and edge length (a) 120, (b) 150, and (c) 225 nm. Only the solid phase is shown (in gray) for clarity.

Nowadays, measuring PSD and pore connectivity in 3D images is well established. The standard way to extracting a PSD from a 3D digital image, is to consider spherical pores and apply the so-called maximal ball algorithm, which finds the largest inscribed sphere centered on each void lattice of the 3D image that just touches the solid boundary.^31^ This method has been successfully employed to obtain PSDs for Vycor,^31,95^ and hence it is also used in the present study. Typical 3D images of Vycor porous glass with sample sizes of 120, 150, and 225 nm (in all cases with a pixel size of 0.75 nm) are presented in Figure 8a–c, respectively.

**Figure 9:**
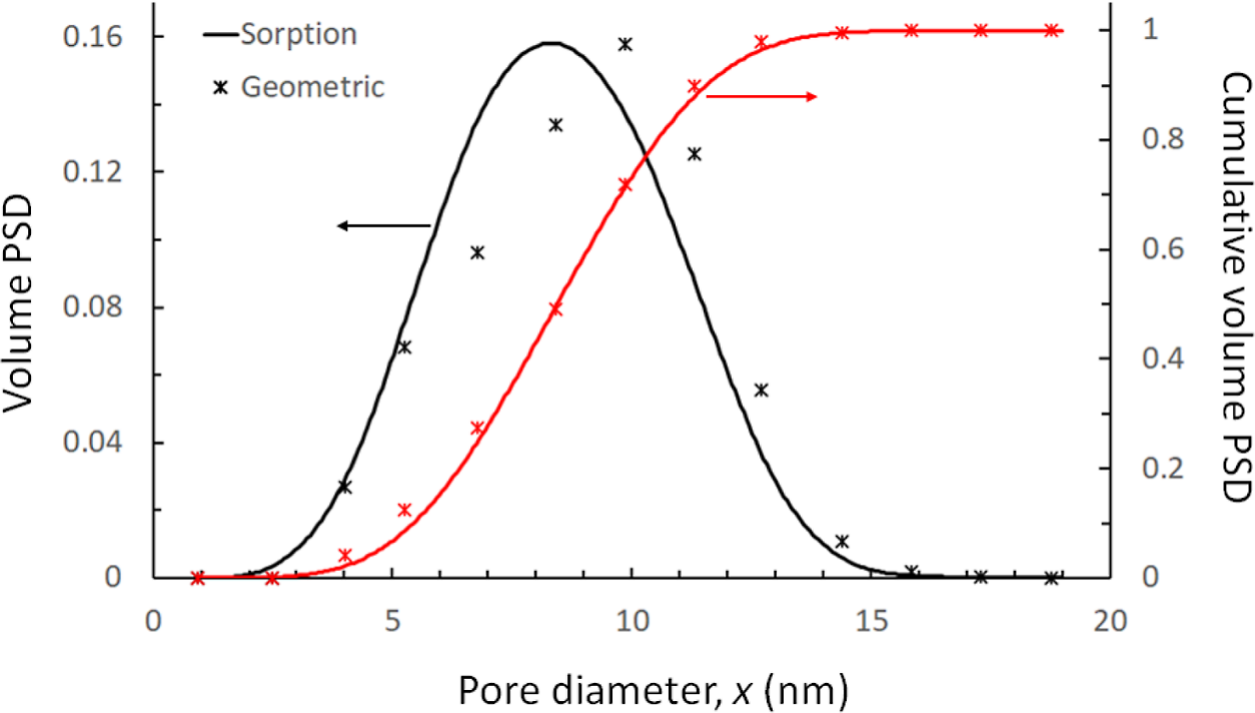
Comparison between geometric PSD measured on 3D digital images of reconstructed Vycor porous glass (pixel size 0.75 nm and edge length 225 nm) and extracted PSD from adsorption–desorption isotherms. Respective cumulative PSDs for each method are also presented using the same symbols, in red.

In Figure 9, we compare the volumetric PSD measured in the 3D reconstructed image of Vycor with the sample size of 225 nm, with the average PSD extracted from the various N_2_ sorption isotherms. It is seen that the two PSDs are in very good agreement, considering the different origins of each method. Note that the geometric PSD is also in excellent agreement with that obtained by Porcheron et al.,^95^ employing the same measurement technique on a similar 3D image of Vycor.

The measurement of pore connectivity, *z*, in 3D structures is less trivial. The basic idea is to first form a skeleton of the 3D image and accordingly measure the connectivity, *z*_*i*_, of each junction *I*, in the skeleton to obtain a distribution of *z* values, from which we can calculate the average connectivity of the pore structure. There are several methods employed for skeletonization, from which we adopt the parallel medial axis thinning of 3D binary images^96–98^ that has been further developed and implemented in MATLAB using the image processing toolbox to quantify the network of cell processes in bones.^99^ Once the skeletal network is formed, it is straightforward to identify the various junctions and perform statistics on junction connectivity values. Note that the medial axis thinning approach for skeletonization of 3D structures has been successfully employed to obtain the pore network connectivity of mesoporous silica adsorbents.^100^

**Figure 10:**
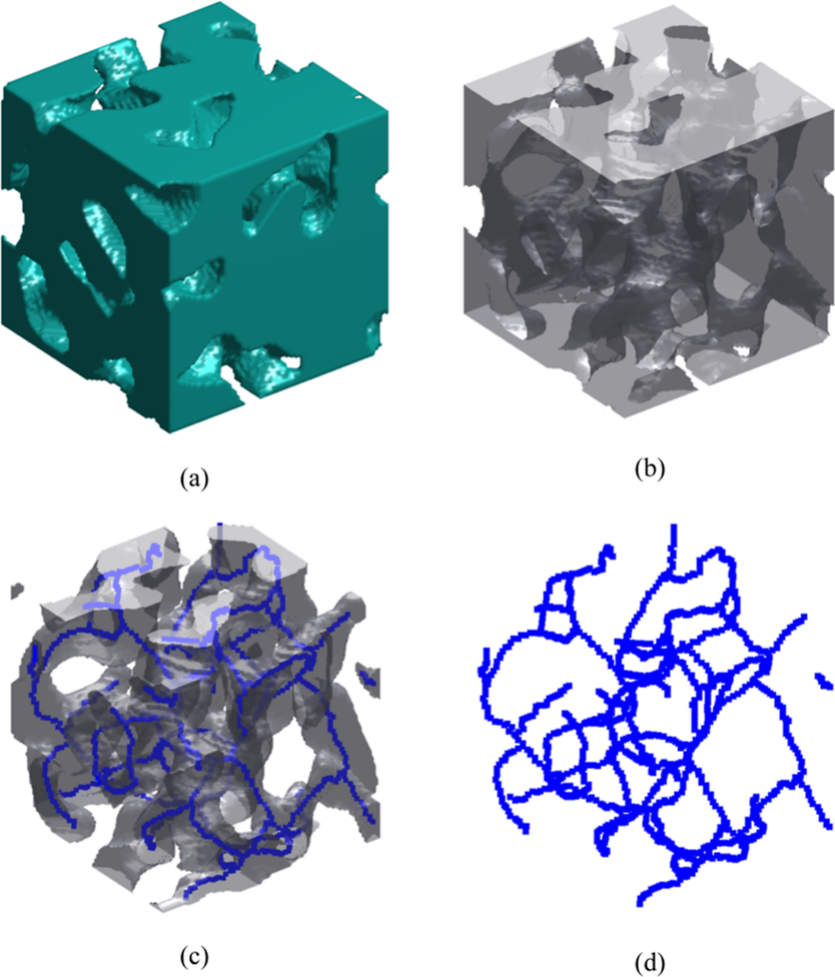
Skeletonization of a 3D digital image of Vycor porous glass with edge length 225 nm. Only the inner core with cube length 60 nm is shown for better visualization. (a) 3D visualization of the inner core sample, where only the solid phase is shown; (b) same sample as in (a), where both phases are shown; (c) same sample as in (a,b), where only the void phase along with its skeletal network (in blue) are shown; and (d) only the skeletal network (in blue) is shown.

The skeletonization process is illustrated in Figure 10, where the inner core (edge length 60 nm) of the 225 nm sample is shown for visualization purposes. In Figure 10a, we present the solid phase of the 3D image only, while in Figure 10b, we present both solid and void phases of the transparent 3D structure. Then, in Figure 10c, we show only the void phase of the image along with its pore skeletal network formed after thinning the void matrix during the skeletonization process. Finally, in Figure 10d, only the pore skeletal network is shown.

**Figure 11:**
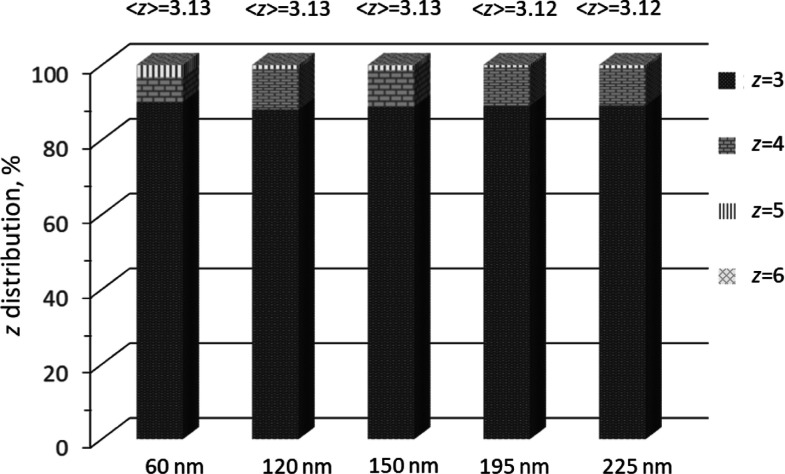
Number distributions of network connectivity, *z*, in skeletal networks of 3D stochastic reconstruction images of Vycor porous glass for various image sizes.

We have applied skeletonization on Vycor images of various sizes and, accordingly, we have measured pore connectivity distributions in each image. Results in terms of *z*-distribution for 3D images of Vycor of various sample sizes (edge lengths) are shown in Figure 11. It is seen that the average value of pore connectivity, ⟨*z*⟩, varies from 3.13 to 3.12 as the sample size increases. This value is in very good agreement with ⟨*z*⟩ = 3.25 extracted from N_2_ sorption experiments. Hence, we conclude that the extracted structural properties of Vycor porous glass from N_2_ sorption isotherms at 77.4 K are in close agreement with those obtained from direct-geometric measurements on realistic 3D digital images of this material.

## 4 Conclusions

We have recently presented a new statistical theory for the adsorption and desorption processes in mesoporous solids in the form of Bethe lattices.^64^ The theory has explicitly accounted for all microscopic mechanisms contributing to the phase change processes in the individual pores composing the pore network. More particularly, capillary-evaporation was controlled by cavitation and gas invasion, while capillary-condensation was controlled by liquid bridging and advanced sorption. In the present work, we apply the new theory to extract accurate structural information on real porous solids by solving the inverse problem in the spirit of our earlier work on the SDCM.^61^ Using Vycor glass as a model porous solid with random pore architecture, we have demonstrated the application of our methodology to extract simultaneously PSD and interconnectivity parameter *z*, reflecting the average number of connections at the junctions of the pore network.

The methodology for solving the complex inverse problem has been presented and tested first on well-defined model pore structures. Hence, we have generated artificial pore networks with different connectivity values, *z* = 3 (Bethe lattice), and predefined PSDs. For these structures, we obtain adsorption–desorption isotherms employing a previously proven algorithmic model^61,64^ and average the results of a statistically significant number of disorder realizations. Accordingly, we employ the resulting sorption isotherms as input in solving the inverse problem to extract PSDs and connectivity parameters *z*, which are found to be in excellent agreement with the predefined PSD and *z* values for each structure.

For the analysis of Vycor porous glass, we employ transition kernels for N_2_ on cylindrical SiO_2_ pores, obtained by modifying BW theory,^22^ using the CLJ 10-4-3 potential developed by Siderius and Gelb,^79^ to account for solid–fluid interactions. The application of the methodology, using N_2_ sorption isotherms measured on Vycor at 77.4 K, results in PSD and *z* values that are notably different from the predictions obtained applying IPM during adsorption and percolation theory during desorption. The extracted morphological properties obtained from analyzing gas physisorption with our developed statistical theory are corroborated by the analysis of realistic 3D digital images of reconstructed Vycor porous glass, showing an excellent agreement between the predictions of the geometric analysis and our predictions.

In the present study, we have considered a Bethe lattice with statistically disordered bonds to approximate the structure of a porous material. Bethe lattices are a special type of pore network that lacks reconnections; thus, analytical expressions for basic percolation probability functions can be obtained. This type of network, although idealized, provides sufficient topological information on the pore structure in terms of network connectivity and pore accessibility and has been used earlier^48,54,101^ in various studies related to Vycor porous glass. Impressively, the results obtained here using this approximation are in a very good agreement with the structural information resulted from 3D stochastic reconstruction. This agreement might be related either to specific properties of Vycor material, e.g., its low porosity, or to the fact that the effects of reconnections existing in real materials are not significant and the present model captures all essential features. Exploring the applicability of the model used here to other porous solids and answering the question stated above is the subject of ongoing research.

Furthermore, it is evident that the methodology presented can be adopted to extract structural information from sorption isotherms of other gas adsorbates, such as Ar, Kr, Xe, He, etc., provided there is available information to determine the respective transition kernels employing BW theory with the CLJ 10-4-3 interaction potential. Hence, it will be interesting to compare structural information (PSD and *z*) extracted from sorption isotherms of different gases on the same Vycor sample, and this will be the subject of future work.

## Supporting Information Available

Algorithmic details on extracting structural properties from sorption isotherms; solution stabilization and smoothing using cubic B-splines; and production of sorption kernels of N_2_ at 77.4 K on cylindrical pores of silica (PDF)
