# Supplementary material for: Gas Sorption Characterization of Porous Materials Employing a Statistical Theory for Bethe Lattices
Source: J Phys Chem A. 2024 May 24;128(22):4573–87. doi: 10.1021/acs.jpca.4c02185 (PMC11163428; doi:10.1021/acs.jpca.4c02185)
Supplement: Supplementary file 1 — jp4c02185_si_001.pdf [file jp4c02185_si_001.pdf]

Gas sorption characterization of porous materials employing a statistical theory for Bethe lattices

Supporting Information

E.S. Kikkinides*

Department of Chemical Engineering, Aristotle University of Thessaloniki, Greece.

D. Enke

Faculty of Chemistry and Mineralogy, Leipzig University, Germany.

R. Valiullin*

Faculty of Physics and Earth System Sciences, Leipzig University, Germany.

*Corresponding author; email: kikki@cheng.auth.gr; valiullin@uni-leipzig.de

Algorithmic details on extracting structural properties from sorption isotherms

To seek a solution of the volume Pore Size Distribution (PSD), $\varphi(x)$, in the adsorption-desorption integral equations, we must first discretize it setting, $\varphi(x) \cong \boldsymbol{\varphi}$, using a certain number of discrete pore sizes, called nodes. Hence, we must first set the limits of integration in pore size, x, from $[0, \infty]$ to a finite region of $[x_{min}, x_{max}]$, with x_{min} and x_{max}, being the respective lower and upper pore sizes of the PSD that we want to extract. Evidently, these values are not known a priori, and a trial-and-error procedure is needed to improve the accuracy and resolution of the extracted PSD.

Since we seek to extract a discrete PSD in $[x_{min}, x_{max}]$, we first replace integrals by sums in Eqs. (5) and (12), which are then written as follows:

$$\theta^{(exp,ads)}(p_i) = \theta_i^{(exp,ads)} = \sum_{j=1}^{N} K_{ij}^{(ads)} \varphi_j \qquad \text{(S1a)}$$

and,

$$\theta^{(exp,des)}(p_i) = \theta_i^{(exp,des)} = \sum_{j=1}^{N} K_{ij}^{(des)} \varphi_j \qquad \text{(S1b)}$$

The above equations can be written for a set of M_a pressure points for adsorption, and M_d pressure points for desorption, with a total number of pressure point, $M=M_a+M_d$. N is the number of discrete cylinder sizes for which we have input kernels. Thus, we employ the following general set of equations, for i=1,M and j=1,N ($N < M$):

$$\theta^{(exp)}(p_i) = \theta_i^{(exp)} = \sum_{j=1}^{N} K_{ij} \cdot \varphi_j \qquad \text{(S2a)}$$

Or equivalently, we need to minimize:

$$r_\theta(\boldsymbol{\varphi}) = \frac{1}{2}\left\|\boldsymbol{\theta}^{(exp)} - \mathbf{K} \cdot \boldsymbol{\varphi}\right\|^2 \qquad \text{(S2b)}$$

with,

$$\theta_i^{(exp)} = \begin{cases} \theta_i^{(exp,ads)} & 1 \le i \le M_a \\ \theta_i^{(exp,des)} & M_a + 1 \le i \le M \end{cases} \qquad \text{(S3a)}$$

and,

$$K_{ij} = \begin{cases} K_{ij}^{(ads)} = a_{ij}^{(ads)} + b_{ij}^{(ads)} + c_{ij}^{(ads)} & 1 \le i \le M_a \\ K_{ij}^{(des)} = a_{ij}^{(des)} + b_{ij}^{(des)} + c_{ij}^{(des)} & M_a + 1 \le i \le M \end{cases} \qquad \text{(S3b)}$$

Parameters $a_{ij}^{(ads)}, b_{ij}^{(ads)}, c_{ij}^{(ads)}, a_{ij}^{(des)}, b_{ij}^{(des)}, c_{ij}^{(des)}$ are determined by the following expressions:

$$a_{ij}^{(ads)} = \begin{cases} \theta_{g_{i,j}} & x_j \le x_n \\ 0 & x_j > x_n \end{cases} \qquad \text{(S4a)}$$

$$b_{ij}^{(ads)} = \begin{cases} \theta_{g_{i,j}} P_{tr,f,i} + \theta_{n_{i,j}}\left(1 - P_{tr,f,i}\right) & x_n < x_j \le x_g \\ 0 & \text{elsewhere} \end{cases} \qquad \text{(S4b)}$$

$$c_{ij}^{(ads)} = \begin{cases} \theta_{n_{i,j}} & x_j > x_g \\ 0 & x_j \le x_g \end{cases} \qquad \text{(S4c)}$$

and,

$$a_{ij}^{(des)} = \begin{cases} \theta_{g_{i,j}} & x_j > x_c \\ 0 & x_j \leq x_c \end{cases} \tag{S4d}$$

$$b_{ij}^{(des)} = \begin{cases} \theta_{g_{i,j}} P_{tr,e,i} + \theta_{c_{i,j}} \left(1 - P_{tr,e,i}\right) & x_g < x_j \leq x_c \\ 0 & \text{elsewhere} \end{cases} \tag{S4e}$$

$$c_{ij}^{(des)} = \begin{cases} \theta_{c_{i,j}} & x_j \leq x_g \\ 0 & x_j > x_g \end{cases} \tag{S4f}$$

where $\theta_{n_{i,j}}$, $\theta_{g_{i,j}}$, $\theta_{c_{i,j}}$, are the various nucleation and growth kernels for condensation and evaporation of N_2 at 77.4 K, in cylindrical pores of silica (SiO2).

Function, $P_{tr,f,i} = P_{tr,f}\left(p_i, z, f, \boldsymbol{\varphi}\right)$, is calculated from:

$$P_{tr,f,i} = 2I^{z-1} - I^{2(z-1)} \tag{S5a}$$

with,

$$I = P_{n,f,i} + \left(P_{g,f,i} - P_{n,f,i}\right) \cdot [(1-f)I]^{z-1} \tag{S5b}$$

and,

$$P_{n,f,i} = P_{n,f}(p_i) = \frac{\left[\sum_{j=1}^{N} x_j^{-2} \cdot \varphi_j\right]_{x_j \leq x_n(p_i)}}{\left[\sum_{j=1}^{N} x_j^{-2} \cdot \varphi_j\right]} \tag{S5c}$$

$$P_{g,f,i} = P_{g,f}(p_i) \cong \frac{\left[\sum_{j=1}^{N} x_j^{-2} \cdot \varphi_j\right]_{x_j \leq x_g(p_i)}}{\left[\sum_{j=1}^{N} x_j^{-2} \cdot \varphi_j\right]} \tag{S5d}$$

Similarly, function $P_{tr,e,i} = P_{tr,e}(p, z, f, \boldsymbol{\varphi})$, is calculated from:

$$P_{tr,e,i} = 1 - (1-f)(1-X)^{2(z-1)} \tag{S8a}$$

with

$$X = P_{n,e,i} + \left(P_{g,e,i} - P_{n,e,i}\right)[f + (1 - f) \cdot (1 - (1 - X)^{z-1})] \qquad \text{(S8b)}$$

and,

$$P_{n,e,i} = P_{n,e}(p_i) \cong \frac{\left[\sum_{j=1}^{N} x_j^{-2} \cdot \varphi_j\right]_{x_j \geq x_c(p_i)}}{\left[\sum_{j=1}^{N} x_j^{-2} \cdot \varphi_j\right]} \qquad \text{(S8c)}$$

$$P_{g,e,i} = P_{g,e}(p_i) \cong \frac{\left[\sum_{j=1}^{N} x_j^{-2} \cdot \varphi_j\right]_{x_j \geq x_g(p_i)}}{\left[\sum_{j=1}^{N} x_j^{-2} \cdot \varphi_j\right]} \qquad \text{(S8d)}$$

From the expressions in eqs. (S4) through (S8) it is evident that the matrices $\mathbf{K}^{(ads)}$ and $\mathbf{K}^{(des)}$ have a complex dependence on relative pressure, p/p_0, as well as on the unknown variables, z, f, and $\boldsymbol{\varphi}$ (through $P_{tr,f}$, $P_{tr,e}$). Hence, eq. (S2) is non-linear in terms on the unknown variables and must be solved employing a triple iterating scheme to determine f, z, and $\boldsymbol{\varphi}$ as follows:

In the first iteration loop, in f, at the kth iteration, $f=f_k$, while in the second iteration loop, in z, at the lth iteration, $z = z_l$. Then we solve eq. (S2) for $f=f_k$,, $z = z_l$, to get the volume PSD, $\boldsymbol{\varphi}$ that minimizes, the residual, $r_\theta(\boldsymbol{\varphi})$. Finally, we come with a set of optimal solutions in PSD, $\boldsymbol{\varphi}$, among which we select the one that gives the minimum, value of $r_\theta(\boldsymbol{\varphi})$ for a variety of values of parameters, $f \in [0, 1]$, and $z \in [2, \infty)$. The above procedure is illustrated schematically in Figure S1:

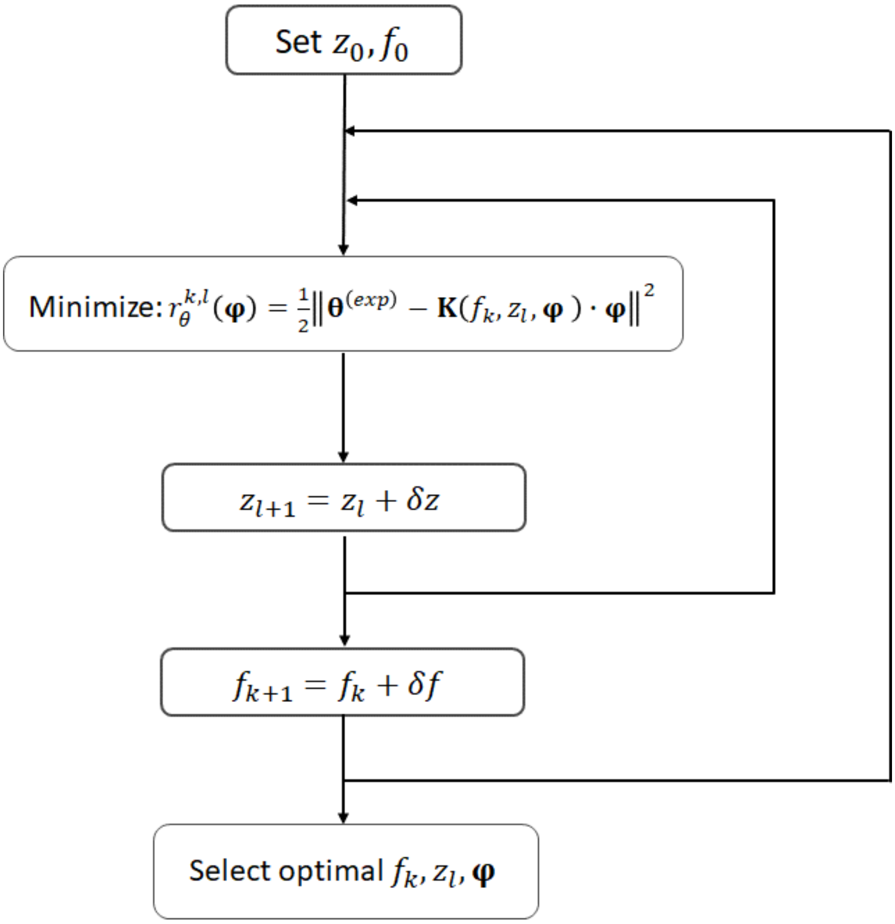

Figure S1. Schematic logical block diagram of the iterative procedure to extract the optimal f, z, $\boldsymbol{\varphi}$, from adsorption-desorption isotherms.

Note that the solution eq. (S2) for a fixed set of values, f=f_k,, $z = z_{l,}$ to get the volume PSD, $\boldsymbol{\varphi}$ is not straightforward, because this equation is non-linear in $\boldsymbol{\varphi}$. Hence, we choose to solve eq. (S2) iteratively, as follows:

1. We start by assigning an initial guess for $\boldsymbol{\varphi}$ employing a random number generator from a uniform number distribution in (0,1).
2. Accordingly, we compute $\mathbf{K}^{(\mathbf{ads})}$, $\mathbf{K}^{(\mathbf{des})}$ from eqs. (S3-S8) and solve the simple linear east square problem: $r_\theta(\boldsymbol{\varphi}) = \frac{1}{2}\left\|\boldsymbol{\theta}^{(exp)} - \mathbf{K} \cdot \boldsymbol{\varphi}\right\|^2$.
3. Then we update to the next iteration using a simple relaxation scheme to avoid the solution from diverging:

$$\varphi_j^{k+1} = w\varphi_j^* + (1-w)\varphi_j^{(k)} \qquad \text{j=1,}N \qquad \text{(S9)}$$

Note that in eq.(S9), we use w=0.5, which is conservative but ensures convergence down to practically machine accuracy.

We repeat steps 2-3, until we achieve converge in **φ** based on the following criterion:

$$L_e = \sqrt{\sum_{j=1}^{N} \left(\varphi_j^{(k+1)} - \varphi_j^{(k)}\right)^2} \leq L_{tol} \qquad j=1,N \qquad \text{(S10)}$$

Note that the elements of the vector of the normalized volume fractions **φ** are subject to the constraints that they should belong to (0,1) and their sum should be equal to one, i.e.: $0 \leq \varphi_j \leq 1 \; \forall j$, $\sum_{j=1}^{N} \varphi_j = 1$.

The above procedure is illustrated in Figure S2, in the form of a logical block diagram:

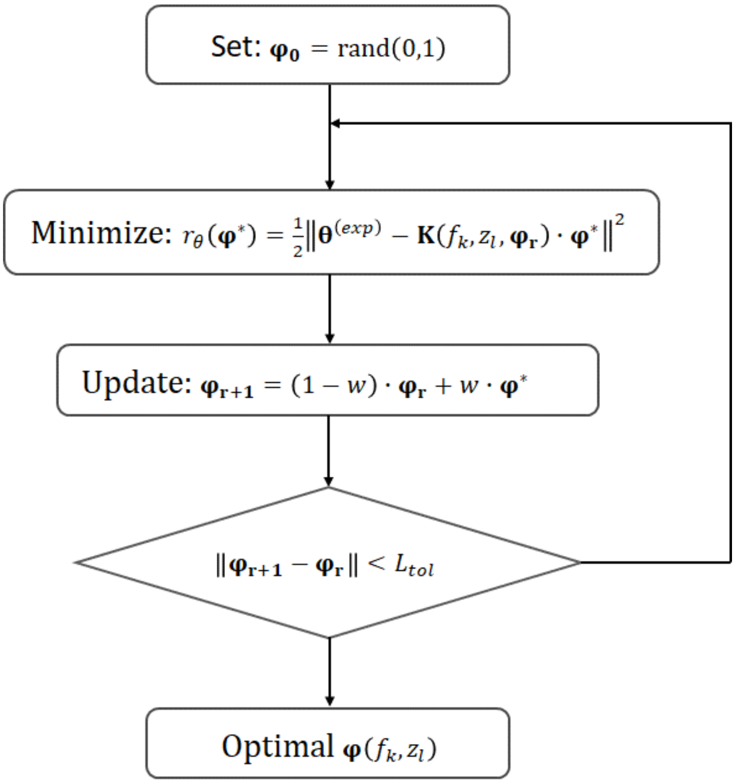

Figure S2. Logical block diagram of the iterative procedure to obtain optimal PSD, **φ**, for a pair of finite size parameter f_k, and pore connectivity, z_l, values.

A routine solving linearly constrained linear least-squares problems based on a two-phase (primal) quadratic programming method has been implemented. The routine has

been successfully employed in extracting PSDs from sorption isotherms in SDCM structures[1]. Issues regarding convergence, sensitivity, and uniqueness of solution, have been considered and checked in a similar fashion with our previous work on SDCM structures[1]. Stringent convergence tolerances have been imposed (accepted error tolerance L_{tol}~1x10^{-12}) and up to 500 iterations have been required in some cases. In all cases the procedure converged to the same final solution for the extracted PSD starting from different initial guesses using the procedure described in step 1, above.

Solution stabilization and smoothing using cubic B-splines

A major drawback of the above methodology is that the number of nodes in pores sizes, N, must be much smaller than the number of points in pressure, M, to get a stable PSD solution[2-3], a constraint that limits the resolution of the PSD and thus the accuracy of the solution. The above issue is resolved by considering the solution $\varphi(x)$ to be represented by piecewise continuous functions, such as cubic B-splines[2-3],

$$\varphi(x) = \sum_{j=1}^{N} s_j \varphi_j \quad \text{(S-11)}$$

where φ_j are the unknown coefficients to be determined, s_j are cubic B-splines with equally spaced knots in the range $[x_{min}, x_{max}]$, and N is now the number of cubic B-splines in the linear combination in Eq.(S-11). Note that each cubic B-spline is a cubic polynomial in four subsequent subintervals between the knots and it is set equal to zero outside this region. This function is non-negative, and its first two derivatives are also continuous [3]. Then, Eq. (S-2a) is written as follows:

$$\theta_i^{k,l} = \sum_{j=1}^{N} K_{ij}^{s}(f_k; z_l; \boldsymbol{\varphi})\varphi_j \quad \text{(S-12a)}$$

where the transformed Kernels using cubic B-splines are written as:

$$K_{ij}^{s} = \int_{x_{min}}^{x_{max}} K_i(x) s_j(x) dx \cong \sum_{k=1}^{N_x} K_{ik} s_{jk} \quad \text{(S-12b)}$$

With N_x being the number of points used to evaluate the integral in Eq.(S-12b), in full accord with the methodology proposed in Ref.[3]. Thereafter, Eqs. (S-12a) and (S-12b)

replace Eq. (S-2a) in the optimization problem used to extract PSD, z, and f from the adsorption-desorption isotherms. The advantage of employing the above procedure is that one can use a very fine resolution in terms of the number of pore sizes, N_x, ensuring a high accuracy in the solution of the various integrals in Eqs.(**Error! Reference source not found.**) and (**Error! Reference source not found.**), while solving the problems for a small number of B-spline nodes[2-3], N, so that $N \ll M$ (but $N_x \gg M$).

Production of Sorption Kernels of N_2 at 77.4 K on Cylindrical Pores of Silica

We follow the method developed by Bonnet and Wolf[4], and later by Morishige[5-6], who have modified the Broekoff and De Boer (BDB) method[7-8], to include solid-fluid interaction at the atomic level, and thermal activation-evaporation to account for activated condensation and cavitation. According to these studies[4-6], and along the framework of a continuum thermodynamic approach, the grand potential of the cylindrical bubble of radius R, per unit pore length, with respect to the filled pore is:

$$\Omega(R) = 2\pi \int_0^R \Delta\rho[\Delta\mu - U_c(r, R_0)]rdr + 2\pi\gamma R \qquad \text{(S13a)}$$

Where $\Delta\rho$ is the difference between bulk liquid and saturation density, $\Delta\mu$ is the difference in the chemical potential between the external vapor in contact with a solid and saturated vapor, γ is the liquid-vapor surface tension (assumed constant and equal to its bulk value), and U_c is the solid-fluid interaction potential for a cylindrical pore. Substituting $\Delta\mu$, by its relation, $\Delta\mu = k_B T \ln\left(\frac{f}{f_0}\right) \cong k_B T \ln\left(\frac{p}{p_0}\right)$, where k_B is the Boltzmann constant, and f/f_0 is the relative fugacity, which is practically equal to relative pressure, p/p_0, for N_2 at T=77.4 K, we get:

$$\Omega(R) = \Delta\rho k_B \ln\left(\frac{p}{p_0}\right)\pi R^2 + 2\pi\gamma R - 2\pi\Delta\rho \int_0^R U_c(r, R_0)rdr \qquad \text{(S13b)}$$

Then we can determine the various characteristic sizes with respect to relative pressure for spontaneous condensation (nucleation), growth (equilibrium), and thermally activated condensation and evaporation (cavitation), applying the various conditions for $\Omega(R)$ according to Bonnet-Wolf (BW) theory[4]. To apply thermally activated

condensation and evaporation mechanisms in BW theory we need to impose an energy barrier, E_c. Following Morishige[5], we set $E_c = 60k_BT$, since this value gives the best fit of critical pore sizes with condensation and cavitation relative pressures for N_2 on KIT-5, an ordered mesoporous silica for which there are molecular simulation results[9]. Details on the implementation of the BW theory can be found elsewhere [4].

From (S13b) we can also determine the statistical film thickness t, by setting $\frac{d\Omega}{dR} = 0$, and finding the root, R_m , of this equation [6] :

$$\Delta\rho \cdot k_B \ln\left(\frac{p}{p_0}\right) + \frac{\gamma}{R_m} - \Delta\rho \cdot U_c(R_m, R_0) = 0 \qquad \text{(S14a)}$$

Then t is determined from the definition:

$$t = R_0 - R \qquad \text{(S14b)}$$

From eqs. (S14a), (S14b) it is evident that the statistical thickness, t, depends on relative pressure p/p_0 and pore radius, R_0, in accord with BDB theory. Consequently, t is employed to determine the various sorption kernels, in terms of the amount adsorbed, q_i, as follows [10-11]:

$$q_i = \begin{cases} V_p \cdot \rho_l \cdot \left[1 - \left(\frac{R_0 - t}{R_0}\right)^2\right] & 0 \le \frac{p}{p_0} \le \left(\frac{p}{p_0}\right)_* \\ V_p \cdot \rho_l & 1 \ge \frac{p}{p_0} \ge \left(\frac{p}{p_0}\right)_* \end{cases} \qquad \text{(S-15)}$$

where $\left(\frac{p}{p_0}\right)_*$ is the respective transition pressure, V_p is the pore volume of the material, and ρ_l is the adsorbate (liquid) density.

The basic difference between the work of Bonnet and Wolf with that of Morishige is the solid-fluid (SiO_2-N_2) interaction potential employed. Bonnet and Wolf have considered the attractive part of the cylindrical Lenard Jones 9-3 (CLJ 9-3) potential, in accord with Saam and Cole's theory[12]. According to this potential, the fluid interacts with a continuum solid of density ρ_s, as follows[13]:

$$U_{c,9-3}(r,R_0) = 2\pi\rho_s\sigma_{sf}^3\varepsilon_{sf}\left[\varphi_6(r,R_0,\sigma_{sf}) - \varphi_3(r,R_0,\sigma_{sf})\right] \quad \text{(S16a)}$$

with

$$\varphi_n(r,R_0,\sigma_{sf}) = \frac{4\sqrt{\pi}}{2n-3}\frac{\Gamma\left(n-\frac{1}{2}\right)}{\Gamma(n)}\left(\frac{\sigma_{sf}}{R_0}\right)^{2n-3}\left[1-\left(\frac{r}{R_0}\right)^2\right]^{3-2n}$$
$$\times F\left[\frac{3-2n}{2},\frac{5-2n}{2};1;\left(\frac{r}{R_0}\right)^2\right] \quad \text{(S16b)}$$

In equations (S16a, S16b), r, is the fluid molecule's distance from the center of the cylindrical pore, R_0, is the pore radius, Γ, is the Gamma function, F, is the hypergeometric function, while σ_{sf}, and ε_{sf}, are the Lenard Jones length and energy parameters for the interaction between the fluid molecules and the solid. Note that n in eq. (S16b) must be either an integer or a half-integer greater than 1/2 [14].

This interaction potential considers pore walls in the form a continuum solid of infinite thickness and predicts a shallow minimum located too close to the cylindrical surface. Evidently, some part of the atomic structure of the solid material must be included to have a faithful representation of the fluid- solid interaction. Thus, Morishige, employed the cylindrical Lenard Jones 10-4 (CLJ 10-4) potential developed by Tjatjopoulos et al.[15], that considers the interaction of the fluid (N_2) with a continuum cylindrical SiO_2 surface with density ρ_A, and zero thickness as follows:

$$U_{c,10-4}(r,R_0) = 2\pi\rho_A\sigma_{sf}^2\varepsilon_{sf}\left[\psi_6(r,R_0,\sigma_{sf}) - \psi_3(r,R_0,\sigma_{sf})\right] \quad \text{(S17a)}$$

with

$$\psi_n(r,R_0,\sigma_{sf}) = 4\sqrt{\pi}\frac{\Gamma\left(n-\frac{1}{2}\right)}{\Gamma(n)}\left(\frac{\sigma_{sf}}{R_0}\right)^{2n-2}\left[1-\left(\frac{r}{R_0}\right)^2\right]^{2-2n}$$
$$\times F\left[\frac{3-2n}{2},\frac{3-2n}{2};1;\left(\frac{r}{R_0}\right)^2\right] \quad \text{(S17b)}$$

Note that all parameters in eq. (S17), including n in eq. (S17b), are defined as previously, except from R_0, which is defined as the distance between the centers of oxygen atoms in the first layer of the pore wall and the pore center[6, 13]. This radius is

related to the (inner) pore radius, R_w, by the expression, $R_0 = R_w + \sigma_{OO}/2$, where σ_{OO}=0.276 nm, is the molecular size for the oxygen atom[6]. Thus, in this case the statistical thickness, t, is determined from the expression[6]:

$$t = R_0 - R = R_w + \sigma_{OO}/2 - R \qquad \text{(S18)}$$

It must be noted that for the case of CLJ 9-3, $R_0 = R_w$ and σ_{00} is set to zero in eq. (S18). This potential has been previously employed in adsorption analysis of MCM-41 type of materials and single-wall carbon nanotubes and in general works well for pores of very small thickness.

A third model, considered in the present work, is the cylindrical Lenard Jones 10-4-3 (CLJ 10-4-3) potential developed by Siderius and Gelb[13], which in the limit of large radii coincides with the celebrated Steele's 10-4-3 potential for planar surfaces[16]. Although Steele's 10-4-3 potential is generally applied only for graphitic structures, the form of the potential was also suggested to be an appropriate representation of fluid-solid interactions involving fcc lattices with exposed (111) and (100) surfaces[17]. Evidently, CLJ 10-4-3 corresponds to an approximate representation of a multi-layered cylindrical pore, where the first layer of atoms is represented by a wall of zero thickness and uniform surface density, and the rest of the solid is integrated starting from a distance $\alpha\Delta$ from the surface, representing a cylinder of uniform volumetric density, keeping only the attractive part of the pairwise potential of the fluid–solid interaction[17]. Then, the resulting interaction potential is written as[13]:

$$U_{c,10-4-3}(r, R_0) = 2\pi\rho_s \Delta \sigma_{sf}^2 \varepsilon_{sf} \Big[\psi_6(r, R_0, \sigma_{sf}) - \psi_3(r, R_0, \sigma_{sf}) - \frac{\sigma_{sf}}{\Delta}\varphi_3(r, R_0 + \alpha\Delta, \sigma_{sf})\Big] \qquad \text{(S19)}$$

where, φ_3, ψ_3, ψ_6, where are defined by eqs. (S16b) and (S17b), respectively, and all other terms are defined as previously. Once again, in this case, $R_0 = R_w$ and σ_{00} is set to zero eq. (S18) to determine the statistical thickness, t.

Parameter α in eq. (S19) was set to α=0.61 by Steele for the case of graphite to improve the accuracy of the model, but it also has theoretical basis, justifying its use for any type

of material. The CLJ 10-4-3 potential is always more strongly adsorbing, exhibits a deeper minimum than the CLJ 10-4 potential and is expected to work better for pores of finite thickness. Finally, the value of thickness Δ, was set to Δ=0.36 nm following[18-19]. This value can be also recovered from the definition, $\Delta=\rho_A/\rho_s$, where ρ_A, ρ_s, are the surface and volume density, respectively, which for silica are ρ_A=15.3 nm^{-2} [20] and ρ_s=44 nm^{-3} [21], giving Δ=0.35 nm.

Note that for the case of N_2 interaction with SiO_2 at 77.4 K, the Lenard Jones length, σ_{sf} , and normalized energy parameter, ε_{sf}/k_B, are [20]: σ_{sf}=0.317 nm , ε_{sf}/k_B=147.25 K. It is evident that the above methodology can be adopted to extract transition kernels for other types of gas adsorbates and solids, provided there is available information on these values.

References

1. Kikkinides, E. S.; Monson, P. A.; Valiullin, R., Sorption Isotherm Reconstruction and Extraction of Pore Size Distributions for Serially Connected Pore Model (Scpm) Structures Employing Algorithmic and Statistical Models. *J Phys Chem C* **2020**, *124*, 21591-21607.
2. Provencher, S. W., A Constrained Regularization Method for Inverting Data Represented by Linear Algebraic or Integral-Equations. *Comput Phys Commun* **1982**, *27*, 213-227.
3. Jagiello, J., Stable Numerical-Solution of the Adsorption Integral-Equation Using Splines. *Langmuir* **1994**, *10*, 2778-2785.
4. Bonnet, F.; Wolf, P. E., Thermally Activated Condensation and Evaporation in Cylindrical Pores. *J Phys Chem C* **2019**, *123*, 1335-1347.
5. Morishige, K., Revisiting the Nature of Adsorption and Desorption Branches: Temperature Dependence of Adsorption Hysteresis in Ordered Mesoporous Silica. *Acs Omega* **2021**, *6*, 15964-15974.
6. Morishige, K., Pore Size Distribution Analysis Using Developing Hysteresis of Nitrogen in the Cylindrical Pores of Silica. *Langmuir* **2022**, *38*, 4222-4233.
7. Broekhoff, J. C.; Deboer, J. H., Studies on Pore Systems in Catalysts .9. Calculation of Pore Distributions from Adsorption Branch of Nitrogen Sorption Isotherms in Case of Open Cylindrical Pores .A. Fundamental Equations. *Journal of Catalysis* **1967**, *9*, 8-+.
8. Broekhoff, J. C.; Deboer, J. H., Studies on Pore Systems in Catalysts .X. Calculations of Pore Distributions from Adsorption Branch of Nitrogen Sorption Isotherms in Case of Open Cylindrical Pores .B. Applications. *Journal of Catalysis* **1967**, *9*, 15-+.
9. Rasmussen, C. J.; Vishnyakov, A.; Thommes, M.; Smarsly, B. M.; Kleitz, F.; Neimark, A. V., Cavitation in Metastable Liquid Nitrogen Confined to Nanoscale Pores. *Langmuir* **2010**, *26*, 10147-10157.

10. Qiao, S. Z.; Bhatia, S. K.; Zhao, X. S., Prediction of Multilayer Adsorption and Capillary Condensation Phenomena in Cylindrical Mesopores. *Microporous and Mesoporous Materials* **2003**, *65*, 287-298.
11. Ustinov, E. A.; Do, D. D.; Jaroniec, M., Equilibrium Adsorption in Cylindrical Mesopores: A Modified Broekhoff and De Boer Theory Versus Density Functional Theory. *J Phys Chem B* **2005**, *109*, 1947-1958.
12. Saam, W. F.; Cole, M. W., Excitations and Thermodynamics for Liquid-Helium Films. *Phys Rev B* **1975**, *11*, 1086-1105.
13. Siderius, D. W.; Gelb, L. D., Extension of the Steele 10-4-3 Potential for Adsorption Calculations in Cylindrical, Spherical, and Other Pore Geometries. *J Chem Phys* **2011**, *135*.
14. Siderius, D. W.; Gelb, L. D., Thermodynamic and Structural Properties of Finely Discretized on-Lattice Hard-Sphere Fluids: Virial Coefficients, Free Energies, and Direct Correlation Functions. *J Chem Phys* **2009**, *131*.
15. Tjatjopoulos, G. J.; Feke, D. L.; Mann, J. A., Molecule Micropore Interaction Potentials. *J Phys Chem-Us* **1988**, *92*, 4006-4007.
16. Steele, W. A., Physical Interaction of Gases with Crystalline Solids .1. Gas-Solid Energies and Properties of Isolated Adsorbed Atoms. *Surf Sci* **1973**, *36*, 317-352.
17. Forte, E.; Haslam, A. J.; Jackson, G.; Müller, E. A., Effective Coarse-Grained Solid-Fluid Potentials and Their Application to Model Adsorption of Fluids on Heterogeneous Surfaces. *Phys Chem Chem Phys* **2014**, *16*, 19165-19180.
18. Gupta, V.; Marc, A. H.; Nguyen, A. V.; Miller, J. D., Crystal Lattice Imaging of the Silica and Alumina Faces of Kaolinite Using Atomic Force Microscopy. *J Colloid Interf Sci* **2010**, *352*, 75-80.
19. Aguilar-Huerta, E.; Cordero-Sánchez, S.; Rojas-González, F.; Villegas-Cortez, J., Description of the Thickness of the Adsorbed Layer, Identification of the Instability Characteristics of the Liquid-Vapour Interface and Assessment of Criticality in Elliptical Pores through the Broekhoff De Boer Theory. *Adsorpt.-J. Int. Adsorpt. Soc.* **2021**, *27*, 1003-1011.
20. Landers, J.; Gor, G. Y.; Neimark, A. V., Density Functional Theory Methods for Characterization of Porous Materials. *Colloids and Surfaces a-Physicochemical and Engineering Aspects* **2013**, *437*, 3-32.
21. Ravikovitch, P. I.; Neimark, A. V., Density Functional Theory Model of Adsorption on Amorphous and Microporous Silica Materials. *Langmuir* **2006**, *22*, 11171-11179.
